# Minimizing Vaccine Wastage in Nigeria: A National Assessment of Vaccine Wastage Rates and Potential Determinants

**DOI:** 10.3390/vaccines12080900

**Published:** 2024-08-08

**Authors:** Kikelomo Lambo, Martha Prescott, Owens Wiwa, Jude Adebowale, Kubura Daradara

**Affiliations:** 1 Clinton Health Access Initiative (CHAI), 7 Ganges Street Off Alvan Ikoku Way, Abuja 900271, Nigeriajudeasolo@yahoo.com (J.A.); 2 Clinton Health Access Initiative (CHAI), 383 Dorchester Ave, Suite 400, Boston, MA 02127, USA; 3 National Primary Health Care Development Agency (NPHCDA), Abuja 900103, Nigeria

**Keywords:** wastage, vaccines, Nigeria, wastage rate, open vial

## Abstract

High vaccine wastage can serve as a critical barrier to achieving the gains of vaccination, especially in a country like Nigeria, where data on vaccine wastage are sparsely available. We determined the country-wide vaccine wastage rates and their determinants through a mixed-methods study conducted across 576 health facilities (primary and secondary) in 24 states in Nigeria. We collected facility-based immunization records from June 2018 to May 2019, in addition to healthcare workers’ interviews and observations of fixed and outreach sessions. The results show that open-vial wastage ranged from 21.2% (95% CI: 20.2%, 22.2%) for the pentavalent vaccine to 72.6% (95% CI: 71.5%, 73.6%) for BCG. Open-vial wastage rates for BCG, measles, and yellow fever vaccines were higher during outreach sessions, with rates of 76.2%, 64.3%, and 65.2%, respectively. For the outreach and fixed sessions, PCV and Penta had the least wastages of 22.0% for fixed sessions and 20.4% for outreach sessions. This study identified vaccine presentation (liquid vs. lyophilized vaccines), vial size (4 dose vs. 5 dose vs. 10 dose vs. 20 dose), RI service delivery strategies (fixed vs. outreach sessions), number of children vaccinated, and human resources (healthcare workers position/cadre) as key determinants of vaccine wastages in Nigeria.

## 1. Introduction

Immunization is widely recognized as the cornerstone of the primary healthcare system and a remarkable success story in global health, averting millions of deaths annually [1]. Between 2000 and 2019, vaccination is estimated to have prevented a minimum of 37 million deaths, representing a 45% decline in fatalities due to 10 vaccine-preventable diseases (VPDs) across 98 low- and middle-income countries (LMICs) when compared with a scenario of no vaccination [2]. While this progress in expanding immunization coverage over the past two decades is commendable, it has been marked by a rise in the cost of a full vaccination course per child due to the introduction of new and more expensive vaccines [3,4]. A situational analysis by the World Health Organization (WHO) showed that global immunization expenditure increased by almost 50% between 2010 and 2017 [4]. This escalating cost of vaccination highlights the critical need for efficient and effective vaccine management, especially in low- and middle-income countries (LMICs) that heavily rely on external assistance for immunization financing. This concern aligns with the current emphasis on transforming immunization systems toward resilience and sustainability.

One of the most significant barriers to achieving efficient immunization is the wastage of vaccines [5]. In 2005, the World Health Organization (WHO) estimated that approximately 50 percent of vaccines produced globally are not administered and hence wasted [6]. However, this is the lone global estimate since then, emphasizing the critical need for updated data and continued efforts to address vaccine wastage. High vaccine wastage rates have a detrimental effect on the availability of life-saving vaccines at service delivery points, undermining global investments to improve vaccine coverage. Vaccines account for a significant quota of immunization costs; thus, there is an imperative need to strengthen global and local vaccine wastage monitoring and ensure wastage is minimized without compromising vaccine coverage [6,7]. The accurate and consistent monitoring of vaccine wastage is essential for improving vaccine forecasting, optimizing supply chain costs, and preventing stock-outs, which may ultimately affect vaccination coverage [8]. However, many low- and middle-income countries (LMICs) lack robust mechanisms to monitor vaccine wastage, particularly at the service delivery level [9].

A study done in 2010 showed that only 19 out of 72 Gavi-eligible countries had available and analyzable data on vaccine wastage at the WHO headquarters [9]. In the absence of country-specific wastage rates, most countries use the WHO indicative generic estimates to forecast and calculate country vaccine needs, which do not usually account for the local context [7]. Several studies examined national vaccine wastage and its determinants in a few African countries [8,10,11]. However, there remains a paucity of data on this subject across the African continent. Several factors, including vaccine characteristics, logistics, national policies, and health worker immunization practices, were shown to influence vaccine wastage in multiple settings [8,10,11,12,13,14,15,16]. Understanding the potential drivers of vaccine wastage and addressing them with context-specific solutions is key for the sustainability of immunization programs, as several countries transition from Gavi support [13].

Nigeria has the second largest population of zero-dose children globally (2.2 million) and has been a beneficiary of technical, managerial, and financial assistance from Gavi, which is the vaccine alliance, to strengthen its immunization system since 2001 [17]. However, Nigeria entered the accelerated phase of the Gavi transition in 2018, with the aim to achieve full self-financing by 2028. As part of this transition, Nigeria is expected to face increased costs for procuring both traditional and new vaccines, where estimates indicate that government vaccine financing needs will rise from $93 million in 2018 to $265 million in 2028 [18]. This transition marks a shift in Nigeria’s financial responsibility for vaccines and highlights the country’s efforts to become self-sustainable in immunization financing. Therefore, identifying opportunities for cost savings within Nigeria’s routine immunization program is imperative, employing measures such as reducing vaccine wastages, introducing the most cost-efficient vaccine, or swapping more costly vaccines for similarly efficacious ones based on antigen presentation. However, there is a dearth of data on the rates and drivers of vaccine wastage rates at the national and sub-national levels in Nigeria. Thus, this study aimed to assess the state-specific vaccine wastage rates and potential determinants for nine selected vaccines.

## 2. Materials and Methods

### 2.1. Study Design and Period

This was a mixed-method, cross-sectional study conducted in 24 states spread across the six geopolitical zones of Nigeria from April 2019 to October 2019.

### 2.2. Study Setting

Nigeria is a country in West Africa and the most populous country in Africa. It is a federal country comprising 36 states that are grouped into six geopolitical zones and a Federal Capital Territory (FCT) [19,20]. According to the National Bureau of Statistics, Nigeria had a projected population of 201 million inhabitants in 2019, with an annual birth cohort of 6.8 million children [21].

Immunization services are delivered in Nigeria through fixed and outreach services and occasionally through mobile services and supplemental immunization activities. In Nigeria, vaccine procurement is done by the United Nations Children’s Fund (UNICEF) and is delivered bundled with devices quarterly. Upon arrival in the country, the vaccines are stored in national/zonal cold stores and distributed quarterly to the states through third-party logistics (3PL) using a PUSH system. Approximately 33,000 functional primary health centers (PHCs) deliver immunization services across the country [22]. 

### 2.3. Study Population and Sampling

**Figure 1:**
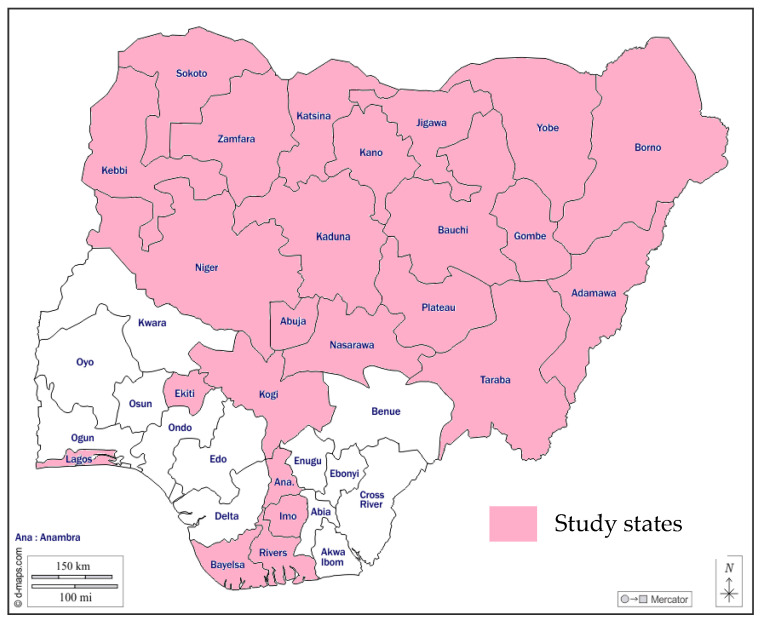
Map of Nigeria showing selected states.

This study included primary and secondary health facilities that provided routine immunization services in Nigeria’s rural and urban areas. Overall, 24 states were purposively selected for assessment by a team that comprised representatives from the National Primary Health Care Development Agency (NPHCDA); Gavi, the Vaccine Alliance; UNICEF; the World Health Organization (WHO); African Field Epidemiology Network (AFENET); and Clinton Health Access Initiative (CHAI). The following states were selected: Borno, Plateau, Bayelsa, Kogi, Adamawa, Nasarawa, Kaduna, Gombe, Niger, Bauchi, Taraba, Kano, Katsina, Kebbi, Zamfara, Yobe, Jigawa, Sokoto, Lagos, Rivers, Imo, Ekiti, Anambra, and the FCT (Figure 1). The Supplementary Materials section contains a summary of the selected states for this study and the justification for selection (Annex S2). 

The maximum sample size was determined mainly by budget and logistical considerations. However, we balanced our need to maximize our resources by ensuring our level of precision around our estimates was acceptable. Using a stratified multi-stage sampling approach, rural and urban health facilities were selected in each state. First, the health facilities within each selected state were stratified into rural and urban categories. To ensure we had a representative sample, the proportionate percentage and number of health facilities to be selected for each category was calculated, and with the aid of a randomizer, the facilities to be assessed were selected in each stratum. A detailed breakdown of the selection by state is found in the Supplementary Materials (Annex S2).

### 2.4. Study Procedures

#### 2.4.1. Data Collection and Management

A total of 192 data collectors and 24 supervisors were hired (8 data collectors and 1 supervisor per state). Before commencing the data collection, national-level supervisor training was conducted, while state-level training of the data collectors occurred in each of the implementing states. The training consisted of walking through the purpose of this study, data collection methods, and ethical considerations. Teams comprising one interviewer and a supervisor participated in the facility assessments. In addition, State Government Officials from the State Primary Health Care Development Agency (SPHCDA) periodically monitored the data collection. Direct supervision within facilities was conducted for all teams on a rolling basis, and troubleshooting support was provided to all enumerators via a pre-determined communication tree, including CHAI staff acting as quality assurance data auditors. Any issues were flagged immediately for the data auditing team to resolve. Data were collected via mobile phones using the Survey CTO software (version 2.60). At the end of each day, each state supervisor worked closely with a CHAI analyst to review the uploaded data and provide feedback to the team. At the end of every week, the research team conducted data quality checks and analyzed the data. 

Data were collected retrospectively and prospectively through 12 months of immunization record reviews, healthcare worker (HCW) interviews, and observations of fixed and outreach sessions.

#### 2.4.2. Immunization Record Reviews

To obtain facility data on immunization session size, data were extracted on fixed and outreach immunization sessions, as recorded in the health facility’s monthly summary immunization form between June 2018 and May 2019. This form contains data on the type of session conducted and the number of children who received each type of vaccine. The data collection period ensured that immunization sessions from two different seasons within a year were collected. To obtain data on the doses opened during an immunization session and any wastage recorded, all data was extracted from the health facility monthly utilization form (VM1a) for the months starting June 2018 through to May 2019 for all selected facilities. 

#### 2.4.3. Healthcare Worker Interviews

Healthcare workers were interviewed in a quiet, private space. Interviews were conducted only after this study was thoroughly explained and informed consent was obtained. At least one EPI healthcare worker was interviewed per facility to collect information on the training status, vaccination knowledge and practices, facility policies, wastage trends, and types of vaccines and services provided.

#### 2.4.4. Observation of Fixed and Outreach Sessions

Data collection at each facility was scheduled by the state supervisor ahead of time such that a fixed immunization session was observed on the day of data collection within the facility. When feasible, outreach sessions were also observed by data collectors. Since outreach sessions are less common and often only occur once a month, a target was set to observe at least two per facility (at least 48 outreach sessions). The data collectors maintained a non-interfering role during the observation sessions, solely focusing on observation. No personal identifying information was collected from the caregivers. Instead, information about the session was gathered, including the initial number of children in the waiting room, start and stop times of each child’s services, administered vaccines per service, the number of doses received by each child, the overall count of opened vials per antigen, and any incidents of vial breakage or damage during the session. At the commencement of the session, healthcare workers informed caregivers about the official observation, but since no personal information regarding the caregivers or children was collected, individual consent was not sought from each caregiver.

### 2.5. Operational Definitions

Estimate of session size: the mean, median, and frequency of the children immunized per session on the observation day (observed) and within the past year (EPI record review).

HCW perspectives on the Multi-Dose Vial Policy (MDVP): Healthcare worker (HCW) knowledge of the minimum number of children for which multi-dose vials should be opened. Healthcare workers’ (HCWs’) knowledge of how unused doses for an opened vial should be handled.

Immunization session frequency: number of times an immunization session was offered in the past month.

Open-vial wastage (for observed sessions):

(1) $$Percentage=\frac{\left(vials\;opened*\#\;doses\;per\;vial\right)-doses\;put\;back\;in\;fridge-children\;immunized}{\left(vials\;opened*\#\;doses\;per\;vial\right)-doses\;put\;back\;in\;fridge}$$

For the vials opened and the number of children vaccinated, the data collector directly observed and recorded this activity during the observed session; for the doses put back into storage, this was asked of the healthcare worker at the end of the session and visually seen by the data collector. The observed facility-specific open-vial wastage between the fixed and outreach sessions was pooled. The average value was then calculated for each facility, and the results were aggregated for the state.

Number of children vaccinated per session: For both the observation and facility record review, we only included sessions where at least one child received the vaccine in question. For both record types, we then created a tally count of the number of children that received any dose of the vaccine regardless of the schedule or age.

Open-vial wastage (data extracted from health facility records): The estimation of the wastage from health facility records was conducted using both the monthly data from the facility immunization summary sheet and the monthly health facility vaccine utilization reporting form. After unreliable values were cleaned out from the facility immunization summary sheet, a facility-specific monthly sum of the number of children vaccinated per vaccine was created for the 12-month period for which data were collected. Similarly, for the utilization records, after unreliable estimates were removed, a monthly summary of the number of doses reported as opened was created for each of the 12 months, from which data were extracted for each facility. The monthly values for each facility were then matched, and in cases where both the number of children vaccinated and the number of doses opened were available, a wastage value was calculated as follows:

(2) $$Wastage\;percentage=\frac{Number\;of\;doses\;opened-Number\;of\;children\;vaccinated}{Number\;of\;doses\;opened}$$

Closed-vial wastage (for observed sessions):

(3) $$Wastage\;percentage=\frac{Number\;of\;total\;vials\;discarded\;during\;observed\;session}{Number\;of\;vials\;at\;facility\;at\;start\;of\;session}$$

Vials were recorded as wasted for any of the following reasons: vaccine vial monitor (VVM) at or beyond the discard point, expired, breakage, freezing, or label removed. For these numbers, the data collector directly observed and recorded this activity during the observed session. For consistency, only those sessions reliable enough to estimate the open-vial wastage were used to estimate the closed-vial wastage.

Overall wastage (observation and records): Percentage of doses that were wasted during a session due to open- and closed-vial wastage combined. For all vaccine wastage indicators, data were collected on nine selected antigens (BCG, OPV, Td, measles, pentavalent, yellow fever, IPV, PCV, and hepatitis B). 

### 2.6. Data Analysis

All data analysis was conducted using Stata version 15 and SPSS version 21. Quantitative variables were summarized using means and standard deviations (SDs), and where appropriate, medians and interquartile ranges (IQRs). Categorical variables, on the other hand, were summarized as frequencies and proportions.

Means or frequencies were reported with their corresponding error estimates (e.g., standard error or 95% confidence interval). All error values were corrected to account for the stratified-multistage clustered sample design. When the strata contained single units, they were treated as certainty units in the error estimation. Wastage estimates were reported per state, and sampling was state representative without the need for weighting. To examine the characteristics according to the potential vaccine presentations, indicators were reported for separate antigens (bOPV, Penta, PCV, and YF) since these vaccines varied in terms of the dose administration, vial size presentation, and multidose vial policy. 

To identify the potential determinants of wastage, the average wastage rates from the session observation were used, as this was the most complete determinant dataset. T-tests were used to compare the average rates across different categories of assessed health facility characteristics. The correlation coefficient was assessed for the relationship between the continuous independent variables and the wastage rates per vaccine. A *p*-value of <0.05 was considered significant.

## 3. Results

Overall, 576 health facilities were included in this study, with 77% (446 facilities) being rural health facilities. The Supplementary Materials contain the profiles of all the selected facilities (Annex S2). Totals of 2304 and 1080 fixed and outreach sessions were observed, respectively (Table 1).

**Figure 2:**
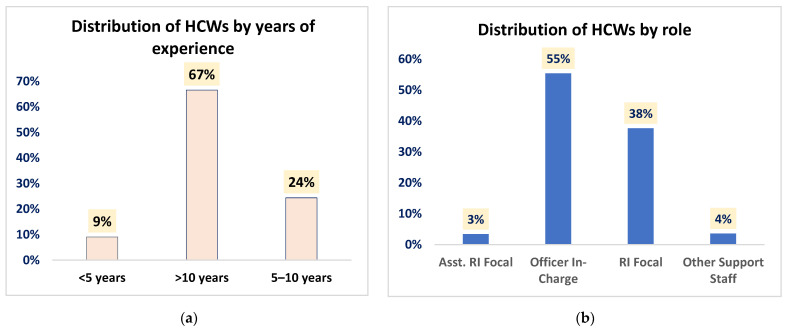
Distribution of healthcare workers by years of experience (**a**) and role (**b**).

In total, 619 HCWs were interviewed, with most being officers in charge (55%) and having greater than 10 years of experience (67%), as shown in Figure 2. In most states (13 of 24), the commonest arrangement for vaccine administration was that involving two HCWs, where one administered while the other recorded, followed by that where one HCW conducted the entire session (7 of 24 states). Only four of these states (Ekiti, Gombe, Rivers, and Yobe) had more than two HCWs present in most sessions. This is summarized in the Supplementary Materials (Annex S1). The storage characteristics and the stability of the vaccines studied in this research was also documented and is shown in the Table 2 below.

### 3.1. Vaccine Wastage Rates

#### 3.1.1. Open-Vial Wastage: Observation

Overall, the open-vial wastage based on observation across fixed and outreach sessions ranged from 21.2% (95% CI: 20.2%, 22.2%) for the pentavalent vaccine to 72.6% (95% CI: 71.5%, 73.6%) for BCG. For the lyophilized vaccines (BCG, measles, and yellow fever), the open-vial wastage rate was significantly higher in the outreach sessions than in the fixed sessions (Table 3). When this was disaggregated by state, it was noted that the Bayelsa and Yobe states recorded the highest or second-highest wastage rates for three or more antigens. Similarly, the Ekiti and Plateau states recorded the lowest or second-lowest wastage rates for three or more antigens (Table 4).

#### 3.1.2. Open-Vial Wastage: Extracted Data from Health Facility Registries

To estimate the wastage using the extracted data, we used both the monthly data from the facility immunization summary sheet and the monthly health facility vaccine utilization reporting form. After cleaning out the unreliable values, we created a facility-specific monthly sum of the number of children vaccinated per vaccine, and the doses reported as opened for the 12-month period for which we collected data. We then matched the monthly values for each facility, and where there were both values for the number of children vaccinated and the number of doses opened, we calculated a wastage value: (number of doses opened—number of children vaccinated)/number of doses opened). We successfully extracted and matched 95.5% of the available monthly summary records to the corresponding vaccine utilization records across 576 facilities and 24 states. The main reason for non-matched records was that the vaccine utilization records for that period were not available. The method by which the HCWs documented vaccine utilization on the monthly utilization summary sheet did not allow us to estimate the open-vial wastage separately for fixed and outreach sessions. Regardless, the estimation from the record extraction appeared to have a higher wastage estimation for all antigens except the lyophilized antigens (BCG, measles, and yellow fever) compared with what we observed.

#### 3.1.3. Closed-Vial Wastage: Observation

In all 24 states, the closed-vial wastage across the nine antigens was observed to be reported in very few health facilities during immunization session observation. However, in the Borno, Ekiti, Katsina, Kebbi, Lagos, and Rivers states, the closed-vial wastage was reported across different antigens in three or more facilities.

#### 3.1.4. Closed-Vial Wastage: Extracted Data from Health Facility Registries

**Figure 3:**
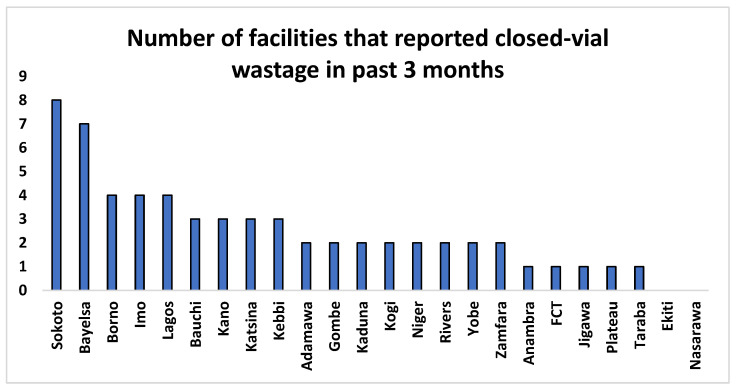
Number of facilities that reported closed-vial wastage in past 3 months.

It was noted that none of the surveyed facilities recorded any closed-vial wastage in the monthly vaccine utilization sheet. However, during the HCW interviews, 10% of healthcare workers indicated they had taken part in closed-vial wastage in the past 3 months that was not documented in the data tools. They attributed these to vaccine vial monitor (VVM) change, expiry, freezing damage, label having been removed, and breakage. Of the 60 health facilities that indicated having closed-vaccine wastage in the past 3 months, the Sokoto and Bayelsa states accounted for the highest proportions of these (see Figure 3).

### 3.2. Potential Determinants of Vaccine Wastage

#### 3.2.1. Vaccine Presentation

The average wastage rates of liquid (OPV, Penta, TT, IPV, PCV, and Hep B) vaccines across the sample total was found to be less (26.5%) than the lyophilized vaccines (BCG, measles, and yellow fever (65.2%)). 

#### 3.2.2. Vial Size

We found a negligible difference between the five-dose and four-dose vials (both averaging approximately 37%). Among the 10-dose vials, there was a large disparity in the wastage rates of the lyophilized and non-lyophilized vaccines, with hepatitis B having a significantly higher wastage rate (61%) than the pentavalent vaccine (37%). On the other hand, there was a minimal difference between the wastage rates of the 10-dose lyophilized vaccines. Similar to the 10-dose vials, the average wastage rate of the 20-dose lyophilized vaccine BCG (68%) was higher than the 20-dose non-lyophilized vaccine OPV (41%).

#### 3.2.3. Mode of Vaccine Administration

We did find evidence that the mode of administration was related to the vaccine wastage rates (*t*-test = 16.916, *p*-value < 0.005). All the vaccines, except for OPV, were administered through injection. OPV was orally given. The average wastage rate of the injectable vaccines was 41.6% and that of the oral (OPV) vaccines was 25.2%. Since OPV is the only non-lyophilized vaccine that was supplied in a size of 20-dose per vial and the only vaccine that was administered orally, there was insufficient ground to conclude that the mode of administration affected the vaccine wastage. 

#### 3.2.4. Number of Children Vaccinated per Session

**Figure 4:**
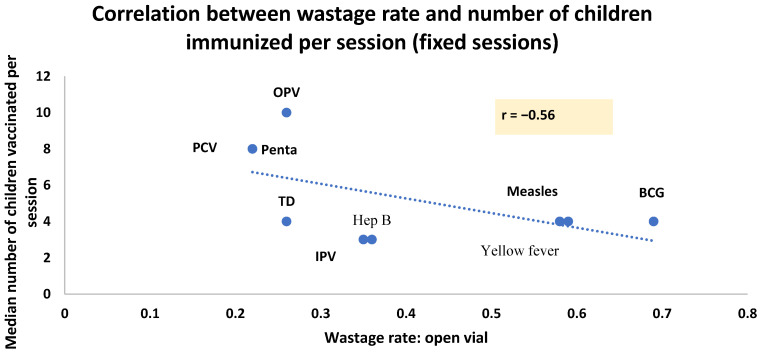
Correlation between wastage rate and number of children immunized per session (fixed sessions).

**Figure 5:**
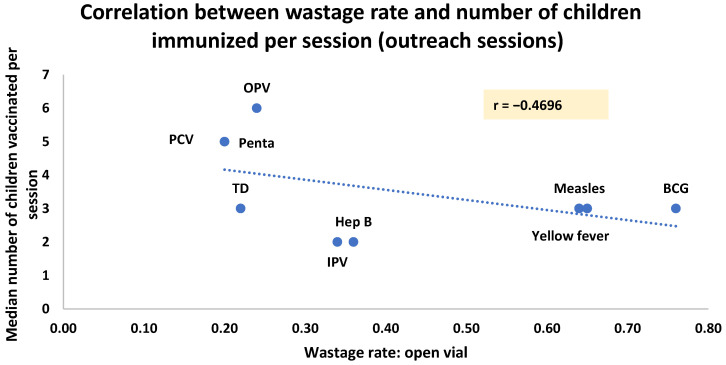
Correlation between wastage rate and number of children immunized per session (outreach sessions).

We noted a negative correlation between the number of children vaccinated and the wastage rates for both the fixed (r = −0.56) and outreach sessions (r = −0.56) (see Figure 4 and Figure 5).

### 3.3. Human Resources

We found that the position of the HCW was associated with the wastage for specific vaccines (BCG *p*-value 0.043; Hep B *p*-value 0.027; measles *p*-value 0.026; TT *p*-value 0.012; yellow fever *p*-value 0.040). The wastage rates were noted to be lower when conducted by an RI focal rather than an assistant RI focal or officer in charge (Table 5).

We also noted that the HCWs with more years of experience (≥10 years) had a significantly lower wastage rate than those with <10 years for some antigens (BCG *p*-value 0.051; Hep B *p*-value 0.038; and measles *p*-value 0.020) (Table 6).

## 4. Discussion

In Nigeria, there is a paucity of dependable countrywide, facility-level data on routine immunization vaccine wastage rates. While a few studies determined wastages in different LGAs and a few states in the country [11], the scope of such reports has been limited and never reflected the country-wide wastage rates burden. In this study, we determined the burden of vaccine wastage and the key enablers of these wastages across selected states in the country’s six geopolitical zones. This is timely, as there is a need to have dependable wastage data to guide forecasting, planning, resource allocation, and health worker capacity building. Such data will also provide pointers to the need to strengthen efficient country-wide supply chain data management systems. Typically, a vaccine may be considered wasted if it is not used to vaccinate eligible children or cohorts due to loss, damage, and/or destruction [7]. For a country like Nigeria, minimizing vaccine wastage is a critical step in ensuring vaccine equity, increasing coverage, and saving costs attributable to the vaccine supply chain. The high cost of the life-saving vaccines’ procurement and supply chain requires that wastages should be minimized, especially as global demands continue to exceed vaccine availability [14]. The determinants of vaccine wastage, such as vaccine administration/presentation, vial size, number of children vaccinated per session, service delivery location, and human resources, explored in this study were previously identified as some of the key factors that influence vaccine wastage [6]. 

In this study, the overall open-vial wastages ranged from 21.2% for Penta and PCV to 72.6% for BCG. The wastage rates for BCG, measles, and yellow fever vaccines were also slightly higher during the outreach sessions compared with the fixed sessions. These wastage rates were higher than the acceptable rate of 50% for BCG, 25% for measles, 5% for PCV, and 10% for OPV [23,24]. This indicates that these wastage rates were not within the acceptable rates for the country. In a previous study conducted in Nigeria, vaccine wastage rates were lower than the findings of this study, as the wastages ranged between 18% and 35% for all vaccines [11]. Inadequacy in the vaccine stock record data and poor monitoring of unopened versus opened vial wastage despite reports that both types of wastage occurred may have been responsible for the lower wastage rate reported in the previous study [11]. Similar to the findings of our study was the report of the study in Bangladesh, which reported higher wastages for BCG (84.9%, range 55–93%) and measles (69.7%, range 28–86%) [16]. In Ethiopia, the vaccine wastage rate was found to be the highest for BCG (55.2%), similar to the findings of the present study [25]. While vaccine wastages are expected and unavoidable in RI service delivery in Nigeria, having wastages within acceptable limits is indicative of effective programming and increased access of eligible children to vaccination [26]. Studies elsewhere in the Littoral Region of Cameroon differed from the findings of the present study, as the wastage rates for BCG ranged between 31 and 33%, while those for OPV and Penta were at 5% [27]. The higher wastage in our study may be attributable to vaccine loss from open vials, poor adherence to the WHO multidose vial policy, and effective utilization of VVM across the supply chain systems. While 94% of the healthcare workers were familiar with the MDVP, only 11% could demonstrate knowledge of the MDVP, indicating limited compliance to the policy. The present study is at variance with a multi-country study conducted in Ghana, Mozambique, and Pakistan, where the wastage rates ranged between 5–33%, with measles reportedly having the highest wastage rates [8]. Moreso, the observed high wastages in our study may also be attributable to the low number of children during RI sessions, along with the observed poor compliance to the multi-dose vial policy. In Nigeria, six vaccines, namely, OPV, Penta, TT, IPV, PCV, and Hep B, were supplied in liquid form while three vaccines—BCG, yellow fever, and measles—were freeze dried or lyophilized vaccines. In this study, the average wastage rates of the liquid form were found to be 29%, in comparison with the lyophilized form, with 65%. Since a lyophilized vaccine needs to be discarded within six hours after re-constitution or immediately after an immunization session, per the MDVP, strengthening demand generation is critical in driving efficient vaccine utilization and uptake. This will increase the vaccination rate during immunization sessions and minimize vaccine wastages, especially for lyophilized vaccines. 

Higher wastages were observed during the outreach sessions compared with the fixed sessions. These findings aligned with the available information in the literature. For instance, in South Sudan, facilities that conducted outreach sessions were reported to have higher wastage rates for liquid vaccines compared with those who did not conduct outreach or mobile RI services [14]. The likelihood of discarding open vials to preserve the potency of vaccines may have been a contributory factor. The liquid vaccines (OPV, Penta, TT, IPV, PCV, and Hep B) had lower wastage rates (26.5%) compared with the lyophilized vaccines (BCG, measles, and yellow fever), with a 65.2% wastage rate. In a previous study in Nigeria, the estimated monthly wastage rates were near 25% for all vaccines and differed little between the liquid and lyophilized vaccine presentations. This indicates that vaccinators could be overly focused on minimizing wastage for lyophilized vaccines but not fully implementing the recommended policies, like the MDVP, to minimize further wastage for liquid vaccines [11]. Elsewhere in Gambia, the wastage rates for lyophilized vaccines (BCG, measles, and yellow fever) were found to be higher than for the liquid vaccines. Higher wastage rates in the lyophilized vaccines were reportedly due to the unused doses at the end of the immunization sessions, while the wastages in liquid vaccines were largely due to expiry and diluent breakage [10]. Another study conducted in Cambodia reported that lyophilized vaccines had higher wastage rates, similar to the findings of the present study compared with liquid vaccines [12]. Elsewhere in South Sudan, liquid vaccines were found to have higher wastage rates, which was likely attributable to a high wastage of unopened vials due to suboptimal vaccine management practices [14].

In some states, higher vaccine wastages were found in rural areas. The rural areas are typically characterized by smaller populations and a weak health system. The findings of the present study also align with previous studies conducted in Iran [28] and India [5], where the vaccine wastage rates were significantly higher in the rural areas compared with the urban areas. Higher vaccination inequities have been documented in rural areas compared with urban areas [29], which is supportive of the increased wastage rate reported in the present study. Elsewhere in Cameroon, a deteriorating power supply was identified as a major factor that contributed to vaccine wastages in rural communities [30]. While this may be applicable in various rural settings, this study did not identify this as a contributor to vaccine wastage, as most healthcare facilities, LGA, and state and national cold stores are dependent on solar, grid, and other alternative power sources (standby generators). Moreso, concerns with power supply at cold stores are quickly escalated and immediately addressed to prevent catastrophic impact [31]. Moreso, while health facilities in Nigeria face enormous power supply challenges despite having some low-capacity generating sets in most facilities to power bulbs and small instruments, the size/capacity of the cold chain equipment for vaccine storage at this level require high voltages to operate them, and thus, the reliance mainly on grid and solar-powered cold chain equipment for vaccine storage.

The cadre/position of the healthcare workers was associated with wastage for specific vaccines (BCG *p*-value 0.043; Hep B *p*-value 0.027; measles *p*-value 0.026; TT *p*-value 0.012; yellow fever *p*-value 0.040). A study conducted in Tanzania reported the role of healthcare workers as likely enablers of higher vaccine wastage rates, which necessitates robust capacity building and compliance with national guidelines for vaccine supply chain management [32]. The inadequate capacity (knowledge and skill) of healthcare workers will inadvertently impact on vaccine management practices. 

The findings of this study show that determining the state-specific wastage rates is a critical component of vaccine management, forecasting, and distribution, and these wastage rates should be considered alongside other key factors and interventions that are beyond the scope of this study, such as country- and state-specific policy frameworks to optimize vaccine supply chain systems. Quantifying the doses and cost implications of vaccine wastage is also important in driving the advocacy for vaccine supply chain strengthening, as such evidence-based analysis of the wastage challenge can clearly demonstrate the implications of high wastage on funding allocations by both government and development partners supporting immunization services. To improve vaccine management and reduce avoidable wastage, each state should develop improvement plans based on their study findings and peculiarity. The data and findings from this study are best used as inputs into the existing state-specific continuous improvement plans and should not be considered as definitively generalizable on their own (e.g., findings should not be used to suggest that all vaccines should have x doses per vial). Additional training, mentoring, and subsequent monitoring through supportive supervision could reduce the amount of unnecessary wastage and result in substantial cost savings for the government. Moreso, the revision and simplification of the existing data tools that healthcare workers currently find to be cumbersome can lead to improved documentation. This study provides a baseline upon which subsequent studies and desk reviews can be compared toward instituting and/or evaluating the effectiveness and efficiency of wastage reduction strategies and interventions.

## 5. Conclusions

This study showed that the wastage of vaccine-preventable diseases is significantly high. While this may negatively impact on the number of children reached with life-saving RI vaccines, there is an inherent economic implication as the country plans to transition from Gavi support. While multi-level effort is required to curb these wastage rates, there is a dire need for the National Primary Health Care Development Agency, in collaboration with the State Primary Health Care Development Agencies and partners, to strengthen the supply chain systems while monitoring effective vaccine utilization across facilities in the country. While this study did not identify cold-chain gaps as a key contributor to the vaccine wastage in this study, effective cold-chain logistics and systems strengthening through replacements and decommissioning of obsolete CCEs, procurement and expansion of cold-chain capacity across the country, and integrated mentorship and supervisory visits (MSVs) to strengthen healthcare workers’ capacity and compliance to the MDVP and other protocol is recommended for improved vaccination and reduced vaccine wastages. Interestingly, with the current national drive to improve accountability and conduct state-specific vaccine forecasting, Nigeria should consider encouraging the periodical inclusion of wastage rate calculations into the routine analysis conducted by EPI managers; this will promote state-specific actions to reduce wastage. 

## Figures and Tables

**Table 1:** A summary of data collection methods by health facility type.

	Immunization Record Review	HCW Interviews	Observed Fixed Sessions	Observed Outreach Sessions
Total (N)	576	576	2304	1080
Urban, n (%)	130 (22.6%)	130 (22.6%)	520 (22.6%)	260
Rural, n (%)	446 (77.4%)	446 (77.4%)	1784 (77.4%)	892

**Table 2:** Storage and stability characteristics.

Vaccine	Presentation per Vial	EPI Program Schedule	Recommended Higher Level Storage (National, Zone, and State)	Recommended Lower Level Storage (LGA and SD/HF)
Bacillus Calmette–Guérin (BCG)	10	4	−25 to −15	+2 to +8
Hepatitis B (Hep B)	20	1	−25 to −15	+2 to +8
Inactivated polio vaccine (IPV)	10	1	+2 to +8	+2 to +8
Measles	10	1	+2 to +8	+2 to +8
Oral polio vaccine (OPV)	10	1	−25 to −15	+2 to +8
Pneumococcal conjugate vaccines (PCV)	4	3	+2 to +8	+2 to +8
Penta	10	3	+2 to +8	+2 to +8
tetanus-diphtheria (TD)	10	2	+2 to +8	+2 to +8
Yellow Fever	10	1	−25 to −15	+2 to +8

**Table 3:** Open-vial wastage rate by antigen.

Vaccine	Overall Open-Vial Wastage Rate (95% CI)	Fixed Session	Outreach Session	*p*-Value
BCG	72.6% (71.5%, 73.6%)	69.00%	76.20%	<0.05
Hep B	35.9% (34.3%, 37.4%)	35.90%	35.80%	≥0.05
IPV	34.6% (33.3%, 36.0%)	35.10%	34.20%	≥0.05
Measles	61.1% (60.0%, 62.1%)	57.80%	64.30%	<0.05
OPV	25.1% (24.1%, 26.1%)	25.90%	24.30%	≥0.05
PCV	21.2% (20.3%, 22.1%)	22.00%	20.50%	≥0.05
Penta	21.2% (20.2%, 22.2%)	22.00%	20.40%	≥0.05
TD	24.1% (22.3%, 25.9%)	25.70%	22.50%	≥0.05
Yellow fever	61.9% (60.9%, 63%)	58.70%	65.20%	<0.05

**Table 4:** Open-vial wastage rate by state.

States	BCG	Hep B	IPV	Measles	OPV	PCV	Penta	TD	YF
Adamawa	41.90%	40.40%	40.60%	59.90%	23.00%	23.40%	24.90%	32%	61.30%
Anambra	56.80%	16.10%	20.50%	51.90%	22.10%	11.10%	14.50%	10.60%	57.10%
Bauchi	73.90%	39.00%	27.80%	56.10%	21.00%	15.80%	19.70%	20%	57.80%
Bayelsa	84.50%	44.70%	43.10%	70.70%	40.30%	23.40%	30.10%	47.80%	74.30%
Borno	67.00%	33.60%	30.00%	69.60%	30.40%	19.30%	25.70%	36.80%	71.50%
Ekiti	77.50%	26.80%	22.30%	50.80%	13.90%	24.80%	12.10%	15.40%	51.10%
FCT	66.50%	25.30%	17.40%	61.30%	19.60%	12.50%	16.20%	29.20%	62.80%
Gombe	43.20%	32.40%	34.70%	64.10%	29.00%	27.30%	29.70%	36.50%	63.30%
Imo	75.30%	17.70%	16.90%	67.80%	15.70%	15.90%	14.40%	16.60%	68.50%
Jigawa	76.10%	19.90%	28.90%	60.30%	21.00%	20.40%	22.90%	15.40%	59.50%
Kaduna	78.20%	40.90%	40.30%	54.90%	23.20%	18.90%	14.60%	14%	54.60%
Kano	80.20%	34.10%	33.60%	71.10%	25.90%	19.40%	18.10%	21.30%	70.80%
Katsina	82.10%	32.70%	35.20%	69.30%	28.30%	17.80%	20.30%	15.40%	71.80%
Kebbi	75.10%	50.70%	41.50%	57.60%	33.70%	23.40%	27.30%	19.50%	57.20%
Kogi	67.40%	39.80%	37.90%	49.90%	20.70%	22.70%	18.70%	21.20%	50.60%
Lagos	68.20%	39.00%	27.40%	55.40%	21.20%	21.50%	20.60%	28.30%	51.80%
Nasarawa	82.30%	46.30%	33.00%	65.30%	25.40%	20.50%	22.00%	29.50%	65.90%
Niger	71.00%	38.80%	39.30%	63.60%	29.30%	21.90%	19.00%	26.70%	62.20%
Plateau	84.10%	11.80%	22.40%	70.60%	17.40%	11.30%	13.90%	19.60%	71.40%
Rivers	70.40%	49.80%	45.50%	49.10%	34.00%	27.70%	24.50%	28.10%	55.50%
Sokoto	80.60%	12.20%	24.80%	68.20%	17.00%	26.50%	15.80%	5%	68.50%
Taraba	74.40%	49.90%	50.90%	58.10%	29.20%	24.50%	22.70%	36.40%	57.80%
Yobe	82.40%	48.50%	55.30%	66.40%	39.50%	43.30%	38.30%	19.10%	66.60%
Zamfara	75.40%	44.00%	47.00%	59.60%	19.90%	18.30%	19.00%	22.10%	59.00%

Green color indicates the lowest or second-lowest vaccine wastage rates for the concerned antigen when compared across states. Red color indicates the highest or second-highest vaccine wastage rates for the concerned antigen when compared across states.

**Table 5:** Association between HCW role and wastage rates.

Vaccines	Officer in Charge (Mean ± SD)	RI Focal (Mean ± SD)	Assistant RI Focal (Mean ± SD)	Other Support Staff (Mean ± SD)	ANOVA	*p*-Value
OPV	34 ± 17	31 ± 16	34 ± 13	30 ± 13	1.143	0.336
BCG	64 ± 21	58 ± 21	65 ± 12	55 ± 15	2.477	0.043
Hep B	54 ± 23	47 ± 22	53 ± 15	50 ± 13	2.774	0.027
IPV	47 ± 20	43 ± 19	54 ± 19	42 ± 16	2.238	0.064
Measles	53 ± 21	47 ± 20	57 ± 17	47 ± 18	2.800	0.026
PCV	27 ± 18	25 ± 18	29 ± 17	23 ± 10	1.110	0.351
Penta	28 ± 16	27 ± 17	32 ± 19	26 ± 13	0.876	0.478
TT	40 ± 21	34 ± 21	48 ± 17	46 ± 17	3.261	0.012
Yellow fever	53 ± 21	47 ± 20	55 ± 17	46 ± 18	2.521	0.040

**Table 6:** Association between HCW experience and wastage rates.

Vaccines	<5 Years (Mean ± SD)	=5 to 10 Years (Mean ± SD)	>10 Years (Mean ± SD)	ANOVA	*p*-Value
OPV	33 ± 15	34 ± 15	32 ± 17	0.906	0.405
BCG	67 ± 16	63 ± 19	60 ± 22	2.998	0.051
Hep B	52 ± 20	56 ± 19	50 ± 24	3.303	0.038
IPV	48 ± 16	47 ± 17	44 ± 21	1.365	0.256
Measles	58 ± 15	53 ± 17	49 ± 22	3.945	0.020
PCV	26 ± 15	27 ± 17	26 ± 18	0.277	0.758
Penta	27 ± 15	30 ± 15	27 ± 17	2.011	0.135
TT	37 ± 22	42 ± 21	37 ± 21	2.363	0.095
Yellow fever	56 ± 14	52 ± 18	49 ± 22	2.655	0.071

## Data Availability

The data presented in this study are available upon request from the corresponding author.

## Supplementary Materials

The following supporting information can be downloaded at: https://www.mdpi.com/article/10.3390/vaccines12080900/s1, Annex S1: Distribution of HCW vaccine administration arrangements by state. Annex S2: Profile of Selected Facilities across states. Annex S3: MODULE B: Health Care Worker Survey.

## References

[1] World Health Organization (WHO) (2020). Agenda 2030: A Global Strategy to Leave No One Behind.

[2] Li X., Mukandavire C., Cucunubá Z.M., Echeverria Londono S., Abbas K., Clapham H.E., Jit M., Johnson H.L., Papadopoulos T., Vynnycky E. (2021). Estimating the Health Impact of Vaccination against Ten Pathogens in 98 Low-Income and Middle-Income Countries from 2000 to 2030: A Modelling Study. Lancet.

[3] Lindstrand A., Cherian T., Chang-Blanc D., Feikin D., O’Brien K.L. (2021). The World of Immunization: Achievements, Challenges, and Strategic Vision for the Next Decade. J. Infect. Dis..

[4] World Health Organization (2020). Situation Analysis of Immunization Expenditure: Key Facts, Working Paper. https://cdn.who.int/media/docs/default-source/immunization/financing/situation-analysis-key-facts.pdf?sfvrsn=8c65d922_2&download=true.

[5] Ministry of Health and Family Health, Government of India (2019). National Vaccine Wastage Assessment.

[6] World Health Organization (2005). Monitoring Vaccine Wastage at Country Level: Guideline for Programme Managers. https://apps.who.int/iris/bitstream/handle/10665/68463/WHO_VB_03.18.Rev.1_eng.pdf.

[7] World Health Organization (2019). Revising Global Indicative Wastage Rates: A WHO Initiative for Better Planning and Forecasting of Vaccine Supply Needs: Concept Note. https://www.who.int/docs/default-source/immunization/tools/revising-wastage-concept-note.pdf?sfvrsn=30e43557_4.

[8] Mvundura M., Ng J., Reynolds K., Theng Ng Y., Bawa J., Bambo M., Bonsu G., Payne J., Chua J., Guerette J. (2023). Vaccine Wastage in Ghana, Mozambique, and Pakistan: An Assessment of Wastage Rates for Four Vaccines and the Context, Causes, Drivers, and Knowledge, Attitudes and Practices for Vaccine Wastage. Vaccine.

[9] Parmar D., Baruwa E.M., Zuber P., Kone S. (2010). Impact of Wastage on Single and Multi-Dose Vaccine Vials: Implications for Introducing Pneumococcal Vaccines in Developing Countries. Hum. Vaccines.

[10] Usuf E., Mackenzie G., Ceesay L., Sowe D., Kampmann B., Roca A. (2018). Vaccine Wastage in The Gambia: A Prospective Observational Study. BMC Public Health.

[11] Wallace A.S., Willis F., Nwaze E., Dieng B., Sipilanyambe N., Daniels D., Abanida E., Gasasira A., Mahmud M., Ryman T.K. (2017). Vaccine Wastage in Nigeria: An Assessment of Wastage Rates and Related Vaccinator Knowledge, Attitudes and Practices. Vaccine.

[12] Wallace A.S., Krey K., Hustedt J., Burnett E., Choun N., Daniels D., Watkins M.L., Soeung S.C., Duncan R. (2018). Assessment of Vaccine Wastage Rates, Missed Opportunities, and Related Knowledge, Attitudes and Practices during Introduction of a Second Dose of Measles-Containing Vaccine into Cambodia’s National Immunization Program. Vaccine.

[13] Dadari I., Ropiti L., Patson A., Okia P., Narasia J., Hare’e T., Namohunu S., Ogaoga D., Gaiofa J., Usuf E. (2022). An Assessment of Vaccine Wastage in the Solomon Islands. PLoS Glob. Public Health.

[14] Bol J., Anyuon N.A., Mokaya E.N. (2021). Assessment of Vaccine Wastage in South Sudan. Pan. Afr. Med. J..

[15] Oberoi S., Mishra P., Gupta V.k., Patnaik S., Garg A., Kaur R. (2021). Vaccine Wastage at Primary, Secondary, and Tertiary Level of Healthcare System—A Study from Northern India. J. Fam. Med. Prim. Care.

[16] Guichard S., Hymbaugh K., Burkholder B., Diorditsa S., Navarro C., Ahmed S., Rahman M.M. (2010). Vaccine Wastage in Bangladesh. Vaccine.

[17] United Nations Children’s Fund (UNICEF) (2023). The State of the World’s Children 2023: For Every Child, Vaccination.

[18] Gavi, The Vaccine Alliance (2018). Successfully Transitioning Nigeria from Gavi Support.

[19] Wong K.L.M., Radovich E., Owolabi O.O., Campbell O.M.R., Brady O.J., Lynch C.A., Benova L. (2018). Why Not? Understanding the Spatial Clustering of Private Facility-Based Delivery and Financial Reasons for Homebirths in Nigeria. BMC Health Serv. Res..

[20] Central Intelligence Agency (2023). The World Factbook.

[21] National Bureau of Statistics (2020). Demographic Statistics Bulletin.

[22] National Primary Health Care Agency (2019). 2020 Nigeria Polio Eradication Emergency Plan.

[23] OLCreate Immunization Module: Vaccine Supply and Stock Management: View as Single Page. https://www.open.edu/openlearncreate/mod/oucontent/view.php?id=53353&printable=1.

[24] Country Planning Cycle Database Nigeria Comprehensive EPI Multi-Year Plan 2011-2015. https://extranet.who.int/countryplanningcycles/planning-cycle-files/nigeria-comprehensive-epi-multi-year-plan-2011-2015.

[25] Mohammed S.A., Wollo University (2022). Vaccine Wastage and Its Contributing Factors in Public Health Facilities, Ethiopia: Explanatory Sequential Mixed Method Design. Community Med. Public Health Care.

[26] Dhage D., Adikane H., Zurmure S. (2017). A Cross-Sectional Study of Assessment of Vaccine Wastage in Tertiary Care Centre of Central India. Int. J. Health Sci. Res..

[27] Nkenyi R., Pak G.D., Tonga C., Chon Y., Park S.E., Kang S. (2022). A Retrospective Review of Vaccine Wastage and Associated Risk Factors in the Littoral Region of Cameroon during 2016–2017. BMC Public Health.

[28] Zahraei S.M., Zamani G., Mohammadbeigi A., Asgarian A., Afrashteh S., Gharibnavaz H., Haghgou M., Kone S. (2020). Estimation of the Wastage Rate of MMR and Pentavalent Vaccines in Open and Closed Vials in Three Western Provinces of Iran. Heliyon.

[29] Naeem M., Khan M.Z.U.I., Adil M., Abbas S.H., Khan M.U., Khan A., Naz S.M. (2011). Inequity in Childhood Immunization between Urban and Rural Areas of Peshawar. J. Ayub Med. Coll. Abbottabad.

[30] Kosari S., Walker E.J., Anderson C., Peterson G.M., Naunton M., Castillo Martinez E., Garg S., Thomas J. (2018). Power outages and refrigerated medicines: The need for better guidelines, awareness and planning. J. Clin. Pharm. Ther..

[31] Ogboghodo E.O., Omuemu V.O., Odijie O., Odaman O.J. (2017). Cold chain management practices of health care workers in primary health care facilities in Southern Nigeria. Pan. Afr. Med. J..

[32] Nestory B., Anasel M., Nyandwi J.B., Asingizwe D. (2022). Vaccine Management Practices among Healthcare Workers in Morogoro, Tanzania: A Cross-Sectional Study. J. Pharm. Policy Pract..

