# Supplementary material for: Minimizing Vaccine Wastage in Nigeria: A National Assessment of Vaccine Wastage Rates and Potential Determinants
Source: Vaccines (Basel). 2024 Aug 8;12(8):900. doi: 10.3390/vaccines12080900 (PMC11359352; doi:10.3390/vaccines12080900)
Supplement: Supplementary file 1 [file vaccines-12-00900-s001.zip › vaccines-3053735-supplementary.pdf]

Supplementary Material

Annex S1. Distribution of HCW vaccine administration arrangements by state.

States	Only One HCW Records and Administers Vaccines	One HCW Records and Another Administers Vaccines	Both HCWs Administer Vaccines	More than Two Health Workers
Adamawa (N=142)	18.3%	41.5%	7%	33.1%
Anambra (N=144)	20.1 %	59.0%	3.5%	17.4%
Bauchi (N=146)	48.6%	41.1%	1.4%	8.9%
Bayelsa (N=151)	12.6%	36.4%	15.9%	35.1%
Borno (N=134)	14.9%	54.5%	1.5%	29.1%
Ekiti (N=145)	6.2%	39.3%	8.3%	46.2%
FCT (N=146)	26 .0%	54.8%	6.2%	13%
Gombe (N=153)	23.5%	28.8%	1.3%	46.4%
Imo (N=143)	12.6%	62.2%	2.1%	23.1%
Jigawa (N=145)	65.5%	22.8%	4.1%	7.6%
Kaduna (N=141)	48.2%	50.4%	0%	1.4%
Kano (N=140)	58.6%	36.4%	1.4%	3.6 %
Katsina (N=145)	65.5%	27.6%	2.1%	4.8%
Kebbi (N=139)	20.9%	68.3%	1.4%	9.4%
Kogi (N=135)	29.6%	57.0%	2.2%	11.1%
Lagos (N=143)	49.0%	36.4%	1.4%	13.3%
Nasarawa (N=141)	39.0%	59.6%	0%	1.4%
Niger (N=141)	15.6%	53.2%	4.3%	27%
Plateau (N=147)	60.5%	27.2%	0%	12.2%
Rivers (N=146)	13.0%	34.9%	13.7%	38.4%
Sokoto (N=154)	46.1%	44.8%	4.5%	4.5%
Taraba (N=128)	19.5%	75.0%	3.1%	2.3%
Yobe (N=138)	11.6%	36.2%	2.2%	50%
Zamfara (N=151)	9.9%	85.4%	0%	4.6%

Green color indicates the most common vaccine administration arrangement in the state.

Annex S2. Profile of Selected Facilities across states.

State	LGA	Facility	Ward	Location	Facility Type
**North Central**					
FCT	Bwari	Chikakore Health Clinic	Bwari	Rural	Primary Health Care Facility
FCT	Gwagwalada	Ikwa Health Clinic	Gwagwalada	Rural	Primary Health Care Facility
FCT	Kuje	PHC Chukuku	Kuje	Rural	Primary Health Care Facility
FCT	Kwali	PHC Dabi/Bako	Kwali	Rural	Primary Health Care Facility
FCT	Bwari	PHC Dutse Alhaji	Bwari	Urban	Primary Health Care Facility
FCT	Bwari	PHC Dutse Makaranta	Bwari	Rural	Primary Health Care Facility
FCT	Kuje	PHC Gbaupe	Kuje	Rural	Primary Health Care Facility
FCT	Municipal Area Council	PHC Gosa	Municipal Area Council	Urban	Primary Health Care Facility
FCT	Kwali	PHC Ike	Kwali	Rural	Primary Health Care Facility
FCT	Municipal Area Council	PHC Jahi	Municipal Area Council	Urban	Primary Health Care Facility
FCT	Municipal Area Council	PHC Jikwoyi	Municipal Area Council	Urban	Primary Health Care Facility
FCT	Bwari	PHC Kawu	Bwari	Rural	Primary Health Care Facility
FCT	Bwari	PHC Kogo	Bwari	Rural	Primary Health Care Facility
FCT	Municipal Area Council	PHC Kpeyegy	Municipal Area Council	Rural	Primary Health Care Facility
FCT	Municipal Area Council	PHC Kurudu	Municipal Area Council	Rural	Primary Health Care Facility
FCT	Kwali	PHC Kwaita	Kwali	Rural	Primary Health Care Facility
FCT	Kwali	PHC Kwali BHC	Kwali	Rural	Primary Health Care Facility
FCT	Bwari	PHC Mpape	Bwari	Rural	Primary Health Care Facility
FCT	Kuje	PHC Pegi	Kuje	Rural	Primary Health Care Facility
FCT	Municipal Area Council	PHC Sheretti	Municipal Area Council	Rural	Primary Health Care Facility
FCT	Municipal Area Council	PHC T/Madaki	Municipal Area Council	Rural	Primary Health Care Facility
FCT	Kuje	PHC Tukpechi	Kuje	Rural	Primary Health Care Facility
FCT	Bwari	PHC Ushafa	Bwari	Rural	Primary Health Care Facility
nFCT	Kwali	PHC Wako	Kwali	Rural	Primary Health Care Facility
Kogi	Lokoja	FSP Felele	Lokoja	Rural	Primary Health Care Facility
Kogi	Okene	FSP Iyarah	Okene	Urban	Primary Health Care Facility
Kogi	Kabba/Bunu	Health Post Bariki	Kabba/Bunu	Rural	Primary Health Care Facility
Kogi	Okene	HP Agassa	Okene	Rural	Primary Health Care Facility
Kogi	Okene	HP Esomi	Okene	Urban	Primary Health Care Facility
Kogi	Ogori/Magongo	HP Obinoyen	Ogori/Magongo	Rural	Primary Health Care Facility
Kogi	Okene	MCH Idoma	Okene	Rural	Primary Health Care Facility
Kogi	Igalamela-Odolu	PHC Agbokete	Igalamela-Odolu	Rural	Primary Health Care Facility
Kogi	Ofu	PHC Aiyede	Ofu	Rural	Primary Health Care Facility
Kogi	Ofu	PHC Ajegwu	Ofu	Rural	Primary Health Care Facility
Kogi	Kabba/Bunu	PHC Aloma	Kabba/Bunu	Rural	Primary Health Care Facility
Kogi	Lokoja	PHC Amuna Ochikala	Lokoja	Urban	Primary Health Care Facility
Kogi	Ofu	PHC Efabo	Ofu	Rural	Primary Health Care Facility

State	LGA	Facility	Ward	Location	Facility Type
Kogi	Kogi	PHC Gegu Beki	Kogi	Rural	Primary Health Care Facility
Kogi	Ijumu	PHC Ikoyi	Ijumu	Rural	Primary Health Care Facility
Kogi	Ofu	PHC Itobe Ogane	Ofu	Rural	Primary Health Care Facility
Kogi	Adavi	PHC Obajana	Adavi	Rural	Primary Health Care Facility
Kogi	Ofu	PHC Ofakaga 11	Ofu	Rural	Primary Health Care Facility
Kogi	Dekina	PHC Ojikpadala	Dekina	Rural	Primary Health Care Facility
Kogi	Adavi	PHC Ojokutu	Adavi	Rural	Primary Health Care Facility
Kogi	Kabba/Bunu	PHC Okebukun	Kabba/Bunu	Rural	Primary Health Care Facility
Kogi	Okene	PHC Okedayo	Okene	Urban	Primary Health Care Facility
Kogi	Ijumu	PHC Osoma	Ijumu	Rural	Primary Health Care Facility
Kogi	Ofu	PHC Umomi	Ofu	Rural	Primary Health Care Facility
Nasarawa	NASARAWA	PHC ADAGBA	O/DGIGA	Rural	Primary Health Care Facility
Nasarawa	LAFIA	PHC AGUDU	WAKWA	Rural	Primary Health Care Facility
Nasarawa	KOKONA	PHC AGWADA	AGWADA	Rural	Primary Health Care Facility
Nasarawa	AKWANGA	PHC AKKAH	AGYAGA	Rural	Primary Health Care Facility
Nasarawa	DOMA	PHC AKPANAJA	AKPANAJA	Rural	Primary Health Care Facility
Nasarawa	NASSARAWA EGGON	PHC ALOGANI	ALOGANI	Rural	Primary Health Care Facility
Nasarawa	KOKONA	PHC ARUME	YELWA	Rural	Primary Health Care Facility
Nasarawa	KOKONA	PHC BOKOKO	KOKONA	Rural	Primary Health Care Facility
Nasarawa	DOMA	PHC DOMA TOWN	SARKIN DAWAKI	Rural	Primary Health Care Facility
Nasarawa	KARU	PHC DUTSEN GOGO	TATTARA-KONDORO	Rural	Primary Health Care Facility
Nasarawa	NASSARAWA EGGON	PHC EVAH	ALUSHI/GINDA	Rural	Primary Health Care Facility
Nasarawa	TOTO	PHC GWARGWADA	GWARGWADA	Rural	Primary Health Care Facility
Nasarawa	LAFIA	PHC IGIBI	MAKAMA	Rural	Primary Health Care Facility
Nasarawa	AWE	PHC JIMI	AZARA	Rural	Primary Health Care Facility
Nasarawa	KEFFI	PHC KAIBO MADA	JIGWADA	Rural	Primary Health Care Facility
Nasarawa	NASSARAWA EGGON	PHC LANGALANGA	IKKA WANGIBI	Rural	Primary Health Care Facility
Nasarawa	KEFFI	PHC MAIN MARKET	YARA	Rural	Primary Health Care Facility
Nasarawa	WAMBA	PHC MARABAN GONGON	GITTA	Rural	Primary Health Care Facility
Nasarawa	KARU	PHC MASAKA	KARU	Urban	Primary Health Care Facility
Nasarawa	DOMA	PHC RUKUBI	RUKUBI	Rural	Primary Health Care Facility
Nasarawa	AKWANGA	PHC UNGWAN DOROWA	NUNKU	Rural	Primary Health Care Facility
Nasarawa	NASSARAWA EGGON	PHC WOLAGA	IKKA WANGIBI	Rural	Primary Health Care Facility
Nasarawa	NASSARAWA EGGON	PHC WULKO	ENDE	Rural	Primary Health Care Facility
Nasarawa	WAMBA	PHC WUNJU	KWARRA	Rural	Primary Health Care Facility
Niger	SHIRORO	BHC GUNU	SHE/GUNU	Rural	Primary Health Care Facility
Niger	MAGAMA	BHC IBETO	IBELU	Rural	Primary Health Care Facility
Niger	KATCHA	BHC KATEREGI	KATAEREGI	Rural	Primary Health Care Facility
Niger	BORGU	BHC WAWA	WAWA	Rural	Primary Health Care Facility
Niger	WUSHISHI	CHC ZUNGERU	ZUNGERU	Rural	Primary Health Care Facility
Niger	MARIGA	CHC BERI	BERI	Rural	Primary Health Care Facility
Niger	GBAKO	CHC FAKUMBA	ETSU AUDU	Rural	Primary Health Care Facility

State	LGA	Facility	Ward	Location	Facility Type
Niger	MARIGA	DURGU HF	BOBI	Rural	Primary Health Care Facility
Niger	AGAIE	EKOSSA MCH/FP	EKOSSA	Rural	Primary Health Care Facility
Niger	BORGU	FAKUN HC	RAFI	Rural	Primary Health Care Facility
Niger	LAPAI	GENERAL HOSPITAL LAPAI	AREWA YAMMA	Rural	Seconday Health Care Facility
Niger	MOKWA	GENERAL HOSPITAL MOKWA	MOKWA CENTRAL	Urban	Seconday Health Care Facility
Niger	RAFI	HF KATAKO	TEGINA CENTRAL	Rural	Primary Health Care Facility
Niger	MAGAMA	MAMBA PHC CLINIC	SALKA	Rural	Primary Health Care Facility
Niger	WUSHISHI	MCH WUSHISHI	SABON GARI	Rural	Primary Health Care Facility
Niger	BIDA	MCH /PPFN	DOKODZA	Urban	Primary Health Care Facility
Niger	SHIRORO	MCH GWADA	EGWA/GWADA	Rural	Primary Health Care Facility
Niger	CHANCHAGA	MCH GWARI ROAD	NASARAWA A	Urban	Primary Health Care Facility
Niger	WUSHISHI	PHC KALIKO	ZUNGERU	Rural	Primary Health Care Facility
Niger	RAFI	PHC GARI GABAS	GUNNA CENTRAL	Rural	Primary Health Care Facility
Niger	MASHEGU	PHC KASANGA	KASANGA	Rural	Primary Health Care Facility
Niger	BOSSO	PHC KODO	KODO	Rural	Primary Health Care Facility
Niger	PAIKORO	PHC PAIKO (TOWN CLINIC)	PAIKO	Rural	Primary Health Care Facility
Niger	MAGAMA	YANGALU PHC CLINIC	YANGALU	Rural	Primary Health Care Facility
Plateau	Bokkos	PHC Bargesh	Bokkos	Rural	Primary Health Care Facility
Plateau	Mangu	PHC Bwonpe	Mangu	Rural	Primary Health Care Facility
Plateau	Bokkos	PHC Chenget	Bokkos	Rural	Primary Health Care Facility
Plateau	Riyom	PHC Clinic -Rahoss	Riyom	Rural	Primary Health Care Facility
Plateau	Bokkos	PHC Danbukur	Bokkos	Rural	Primary Health Care Facility
Plateau	Qua'an Pan	PHC Dokankasuwa	Qua'an Pan	Rural	Primary Health Care Facility
Plateau	Mangu	PHC Doss	Mangu	Rural	Primary Health Care Facility
Plateau	Langtang South	PHC Gamakai	Langtang South	Rural	Primary Health Care Facility
Plateau	Barkin Ladi	PHC Heipang	Barkin Ladi	Rural	Primary Health Care Facility
Plateau	Pankshin	PHC Janaret	Pankshin	Rural	Primary Health Care Facility
Plateau	Mangu	PHC Jannaret	Mangu	Rural	Primary Health Care Facility
Plateau	Mangu	PHC Kondigohor	Mangu	Rural	Primary Health Care Facility
Plateau	Jos North	PHC Lamingo	Jos North	Urban	Primary Health Care Facility
Plateau	Bassa	PHC Mabudi	Bassa	Rural	Primary Health Care Facility
Plateau	Bokkos	PHC Mandar Ken	Bokkos	Rural	Primary Health Care Facility
Plateau	Qua'an Pan	PHC Mere	Qua'an Pan	Rural	Primary Health Care Facility
Plateau	Langtang South	PHC Padaman Shanu	Langtang South	Rural	Primary Health Care Facility
Plateau	Bassa	PHC Pan-Dauda	Bassa	Rural	Primary Health Care Facility
Plateau	Langtang North	PHC Pangna	Langtang North	Rural	Primary Health Care Facility
Plateau	Jos East	PHC Rizek	Jos East	Rural	Primary Health Care Facility
Plateau	Qua'an Pan	PHC Sabon Gamji	Qua'an Pan	Rural	Primary Health Care Facility
Plateau	Riyom	PHC Ta-Hoss	Riyom	Rural	Primary Health Care Facility
Plateau	Qua'an Pan	PHC Tim	Qua'an Pan	Rural	Primary Health Care Facility
Plateau	Pankshin	PHC Town Clinic	Pankshin	Rural	Primary Health Care Facility
**North East**					

State	LGA	Facility	Ward	Location	Facility Type
Adamawa	Yola North	Bachure Phc	Karewa	Urban	Primary Health Care Facility
Adamawa	Demsa	Demsa PHC	Demsa	Rural	Primary Health Care Facility
Adamawa	Demsa	Dilli HC	Dilli	Rural	Primary Health Care Facility
Adamawa	Demsa	Farai Hp	Demsa	Rural	Primary Health Care Facility
Adamawa	Girei	Federal Housing Maternity And Child Health Clinic	Dakri	Rural	Primary Health Care Facility
Adamawa	Girei	Girei A phc	Girei 1	Rural	Primary Health Care Facility
Adamawa	Girei	Girei B Primary Health Centre	Girei 2	Rural	Primary Health Care Facility
Adamawa	Mubi South	LAMORDE	Mubi	Rural	Primary Health Care Facility
Adamawa	Mubi South	lokuwa	mubi	Rural	Primary Health Care Facility
Adamawa	Fufore	Mainne H c	Mainne	Rural	Primary Health Care Facility
Adamawa	Yola North	Major Aminu Health Clinic	Doubeli	Urban	Primary Health Care Facility
Adamawa	Mayo-Belwa	Mallam Babba Township Clinic	Mbilla	Rural	Primary Health Care Facility
Adamawa	Mubi north	Muchalla Model	Muchalla	Rural	Primary Health Care Facility
Adamawa	Mubi South	Munduva B Health Facility	Gude	Rural	Primary Health Care Facility
Adamawa	Mayo-Belwa	Ndivam Njumtobe Phc	Ndikong	Rural	Primary Health Care Facility
Adamawa	Girei	Njobbore Phc	Modire	Rural	Primary Health Care Facility
Adamawa	NUMAN	NUMAN 1 PHC	NUMAN 1	Rural	Primary Health Care Facility
Adamawa	Demsa	Old Demsa HC	Demsa	Rural	Primary Health Care Facility
Adamawa	Fufore	Pariya Phc	Pariya	Rural	Primary Health Care Facility
Adamawa	Numan	Pilotara Phc	Numan 3	Rural	Primary Health Care Facility
Adamawa	Demsa	Primary Health Care Center Kpasham	Kpasham	Rural	Primary Health Care Facility
Adamawa	Girei	Vonukulang Phc	Vonukulang	Rural	Primary Health Care Facility
Adamawa	Yola South	wuru jabbe	Namtari	Urban	Primary Health Care Facility
Adamawa	Yola North	Yelwa Health Clinic	Yelwa	Urban	Primary Health Care Facility
Bauchi	DARAZO	Aisha Isa Yuguda Under 5 Clinic	DARAZO EAST	Urban	Primary Health Care Facility
Bauchi	ZAKI	Ari PHC	TASHENA	Rural	Primary Health Care Facility
Bauchi	KATAGUM	Bulkachuwa PHC	BULKACHUWA	Rural	Primary Health Care Facility
Bauchi	GIADE	Chinkani PHC	CHINKANI	Rural	Primary Health Care Facility
Bauchi	DAMBAM	Dambam Town Maternity	DAMBAM A	Rural	Primary Health Care Facility
Bauchi	KATAGUM	Dandango PHC	MADANGALA	Rural	Primary Health Care Facility
Bauchi	JAMA'ARE	DURR MPHC	DOGON JEJI C	Rural	Primary Health Care Facility
Bauchi	BAUCHI	DURUM PHC	KUNDUN DURUM	Rural	Primary Health Care Facility
Bauchi	WARJI	GOLOLO PHC	KATANGA	Rural	Primary Health Care Facility
Bauchi	GAMAWA	GONGO MHC	GOLOLO SOUTH	Rural	Primary Health Care Facility
Bauchi	ALKALERE	GWARAM PHC	GWARAM A	Rural	Primary Health Care Facility
Bauchi	BOGORO	GYARA PHC	GYARA	Rural	Primary Health Care Facility
Bauchi	DARAZO	KANYA Health Post	DARAZO WEST	Rural	Primary Health Care Facility
Bauchi	DASS	KATANGA Health Post	DURR	Rural	Primary Health Care Facility

State	LGA	Facility	Ward	Location	Facility Type
Bauchi	KIRFI	KUMBI Health Post	BADARA	Rural	Primary Health Care Facility
Bauchi	BAUCHI	MADANGALA	DAN DANGO	Rural	Primary Health Care Facility
Bauchi	TORO	MAGAMA GUMAU	TORO	Urban	Primary Health Care Facility
Bauchi	ITAS GADAU	MASHEMA PHC	MASHEMA	Rural	Primary Health Care Facility
Bauchi	MISAU	MISAU TOWN MATERNITY	KUKADI B	Urban	Primary Health Care Facility
Bauchi	ALKALERE	PALI PHC	PALI EAST	Rural	Primary Health Care Facility
Bauchi	NINGI	SAKWA PHC	ARI	Rural	Primary Health Care Facility
Bauchi	GANJUWA	SORO PHC	GANJUWA B	Rural	Primary Health Care Facility
Bauchi	TAFAWA-BALEWA	Tafawa Balewa MCH	WAI B	Urban	Primary Health Care Facility
Bauchi	SHIRA	ZUBO PHC	ZUBO	Rural	Primary Health Care Facility
Borno	Konduga	707 Estate Clinic	Konduga	Rural	Primary Health Care Facility
Borno	Maiduguri	Bulabulin Dispensary	Maiduguri	Urban	Primary Health Care Facility
Borno	Maiduguri	CAN Centre IDP Camp Clinic	Maiduguri	Urban	Primary Health Care Facility
Borno	Konduga	chabbal clinic	Konduga	Rural	Primary Health Care Facility
Borno	Maiduguri	Fatima Ali Sheriff PHC	Maiduguri	Urban	Primary Health Care Facility
Borno	Maiduguri	GAMBORU MALE DISP.	Maiduguri	Urban	Primary Health Care Facility
Borno	Maiduguri	GAMBORU MARKET DISP.	Maiduguri	Urban	Primary Health Care Facility
Borno	Askira-Uba	Giwi Dispensary	Askira-Uba	Rural	Primary Health Care Facility
Borno	Maiduguri	GWANGE PHC	Maiduguri	Urban	Primary Health Care Facility
Borno	Askira-Uba	Husara Health Center	Askira-Uba	Rural	Primary Health Care Facility
Borno	Konduga	Kofa IDP Camp Clinic	Konduga	Rural	Primary Health Care Facility
Borno	Konduga	Konduga MCH	Konduga	Rural	Primary Health Care Facility
Borno	Maiduguri	Maiduguri Epidemiology Unit Clinic	Maiduguri	Urban	Primary Health Care Facility
Borno	Askira-Uba	Muffa Dispensary	Askira-Uba	Rural	Primary Health Care Facility
Borno	Maiduguri	Njimtilo clinic	Maiduguri	Urban	Primary Health Care Facility
Borno	Maiduguri	PHC Bulabulin Ngarannam	Maiduguri	Rural	Primary Health Care Facility
Borno	Konduga	PHC DALORI	Konduga	Rural	Primary Health Care Facility
Borno	Konduga	PHC Gwange (Dispensary)	Konduga	Rural	Primary Health Care Facility
Borno	Konduga	PHC Jakana	Konduga	Rural	Primary Health Care Facility
Borno	Maiduguri	Ramat Clinic	Maiduguri	Urban	Primary Health Care Facility
Borno	Maiduguri	Teachers Village IDP Camp Clinic	Maiduguri	Urban	Primary Health Care Facility
Borno	Jere	Tungushe Health Clinic	Jere	Urban	Primary Health Care Facility
Borno	Askira-Uba	Uba Dispensary	Askira-Uba	Rural	Primary Health Care Facility
Borno	Maiduguri	Yerwa MCH	Maiduguri	Urban	Primary Health Care Facility
Gombe	Nafada	Barwo Nasarawo PHC	Barwo Nasarawo	Rural	Primary Health Care Facility
Gombe	Nafada	Barwo Winde PHC	Barwo Winde	Rural	Primary Health Care Facility
Gombe	Akko	Bogo Model PHCC	Garko	Rural	Primary Health Care Facility

State	LGA	Facility	Ward	Location	Facility Type
Gombe	Akko	Chilo PHC	Kalshingi	Rural	Primary Health Care Facility
Gombe	Kwami	DOHO PHC	Doho	Rural	Primary Health Care Facility
Gombe	Gombe	Gabukka PHC	Pantami	Urban	Primary Health Care Facility
Gombe	Kwami	GADAM PHC CLINIC	Gadam	Rural	Primary Health Care Facility
Gombe	Akko	Hammadu Kafi PHC	Garko	Rural	Primary Health Care Facility
Gombe	Gombe	Kumbiya Kumbiya PHC	Kumbiya Kumbiya	Urban	Primary Health Care Facility
Gombe	Akko	Kumo PHCC	Kumo Centre	Rural	Primary Health Care Facility
Gombe	Gombe	London PHC	Shamaki	Urban	Primary Health Care Facility
Gombe	Kwami	M/SIDI PHC	Malam Sidi	Rural	Primary Health Care Facility
Gombe	Gombe	Madaki CHC	Bolari East	Urban	Primary Health Care Facility
Gombe	Funakaye	Mutuke Mat	Jillahi	Rural	Primary Health Care Facility
Gombe	Nafada	Nafada PHC	Nafada East	Rural	Primary Health Care Facility
Gombe	Balanga	PHC Bambam	Bambam	Rural	Primary Health Care Facility
Gombe	Billiri	PHC KENTENGERE	Bare	Rural	Primary Health Care Facility
Gombe	Billiri	PHC SANSANI	Billiri North	Rural	Primary Health Care Facility
Gombe	Yamaltu/Deba	Primary Health Care Deba	Deba	Rural	Primary Health Care Facility
Gombe	Yamaltu/Deba	Primary Health care Kwadon	Kwadon/Liji/Kurba	Rural	Primary Health Care Facility
Gombe	Yamaltu/Deba	Primary Health Clinic Liji	Kwadon/Liji/Kurba	Rural	Primary Health Care Facility
Gombe	Funakaye	Ribadu PHC	Ribadu	Rural	Primary Health Care Facility
Gombe	Kwami	SHONGO MATERNITY	Konfulata	Rural	Primary Health Care Facility
Gombe	Funakaye	Town Mat. Bajoga	Bajoga West	Rural	Primary Health Care Facility
Taraba	WUKARI	AL IMAN PHCC	HOSPITAL	Rural	Primary Health Care Facility
Taraba	ARDO KOLA	First Referral H Sunkani	BAKIN DUTSE	Rural	Primary Health Care Facility
Taraba	GASSOL	FIRST REFERRAL HOSP.M/BIYU	GUNDUMA	Rural	Primary Health Care Facility
Taraba	WUKARI	HEALTH CLINIC WAPAN NGHAKU	HOSPITAL	Rural	Primary Health Care Facility
Taraba	KARIM LAMIDO	KARIM PHCC	KARIM A	Urban	Primary Health Care Facility
Taraba	JALINGO	KUNGANA PHCC	TURAKI A	Urban	Primary Health Care Facility
Taraba	GASSOL	MM PHC	MUTUM-BIYU A	Rural	Primary Health Care Facility
Taraba	ZING	Nbosung PHCC	BUBONG	Rural	Primary Health Care Facility
Taraba	JALINGO	NEW BARADE PHC	BARADE	Rural	Primary Health Care Facility
Taraba	WUKARI	NPI General Hospital Wukari	HOSPITAL	Rural	Seconday Health Care Facility
Taraba	JALINGO	PHC MAYO GWAI	SINTALI B	Urban	Primary Health Care Facility
Taraba	GASSOL	PHC MUTUM-BIYU	MUTUM-BIYU B	Urban	Primary Health Care Facility
Taraba	LAU	PHCC ABBARE	ABBARE	Rural	Primary Health Care Facility
Taraba	IBI	PHCC GINDIN WAYA	RIMI UKU II	Rural	Primary Health Care Facility
Taraba	BALI	PHCC GUNDUMA	SUNTAI	Rural	Primary Health Care Facility
Taraba	IBI	PHCC IBI	NWONYO I	Rural	Primary Health Care Facility
Taraba	LAU	PHCC JIMLARI	JIMLARI	Rural	Primary Health Care Facility
Taraba	LAU	PHCC KUNINI	KUNINI	Urban	Primary Health Care Facility

State	LGA	Facility	Ward	Location	Facility Type
Taraba	GASHAKA	PHCC PANWAI	GAYAM	Rural	Primary Health Care Facility
Taraba	ZING	PHCC ZING	ZING	Rural	Primary Health Care Facility
Taraba	LAU	SABONGARI PHCC	MAYOLOPE	Rural	Primary Health Care Facility
Taraba	YORRO	SHOMPA GANAH PHC	KAJONG	Rural	Primary Health Care Facility
Taraba	GASSOL	TURA KEKE PHC	NAMNAI	Rural	Primary Health Care Facility
Taraba	JALINGO	WURO MUSA PHC	KACHALLA SEMBE	Rural	Primary Health Care Facility
Yobe	Tarmua	GARGA HP	Tarmua	Rural	Primary Health Care Facility
Yobe	Damaturu	HC Dikkumari	Damaturu	Rural	Primary Health Care Facility
Yobe	Damaturu	HC Gambir	Damaturu	Rural	Primary Health Care Facility
Yobe	Jakusko	HC Katangana	Jakusko	Rural	Primary Health Care Facility
Yobe	Tarmua	HC Koriyel	Tarmua	Rural	Primary Health Care Facility
Yobe	Damaturu	HC Murfa	Damaturu	Urban	Primary Health Care Facility
Yobe	Tarmua	HC Shekau	Tarmua	Rural	Primary Health Care Facility
Yobe	Geidam	HC State Low cost	Geidam	Urban	Primary Health Care Facility
Yobe	Jakusko	HC Tajuwa	Jakusko	Rural	Primary Health Care Facility
Yobe	Potiskum	HC Zinzano	Potiskum	Urban	Primary Health Care Facility
Yobe	Tarmua	HP Biriri	Tarmua	Rural	Primary Health Care Facility
Yobe	Damaturu	Malum Matari	Damaturu	Urban	Primary Health Care Facility
Yobe	Potiskum	MCH Bula	Potiskum	Rural	Primary Health Care Facility
Yobe	Fika	MCH Fika	Fika	Rural	Primary Health Care Facility
Yobe	Nangere	PHC Dagare	Nangere	Rural	Primary Health Care Facility
Yobe	Fika	PHC Kukar Gadu	Fika	Rural	Primary Health Care Facility
Yobe	Damaturu	PHC Kukareta	Damaturu	Urban	Primary Health Care Facility
Yobe	Damaturu	PHC Mairi	Damaturu	Urban	Primary Health Care Facility
Yobe	Nangere	PHC New Nangere	Nangere	Rural	Primary Health Care Facility
Yobe	Potiskum	PHC Potiskum	Potiskum	Urban	Primary Health Care Facility
Yobe	Tarmua	PHCC Babbangida	Tarmua	Rural	Primary Health Care Facility
Yobe	Jakusko	PHCC Gasamu	Jakusko	Rural	Primary Health Care Facility
Yobe	Potiskum	PHCC Gwange	Potiskum	Urban	Primary Health Care Facility
Yobe	Damaturu	PHCC Maisandari	Damaturu	Rural	Primary Health Care Facility
**North West**					
Jigawa	Abunabo	Abunabo MPHC	Guri	Rural	Primary Health Care Facility
Jigawa	Ahoto	Ahoto MPHC	Buji	Rural	Primary Health Care Facility
Jigawa	Amaryawa	Amaryawa MPHCC	Roni	Rural	Primary Health Care Facility
Jigawa	Auyakayi	Aukayi BHC	Auyo	Rural	Primary Health Care Facility
Jigawa	kiyako	Bamaina PHC	Birnin Kudu	Rural	Primary Health Care Facility
Jigawa	Bekarya	Bekarya BHC	Gumel	Rural	Primary Health Care Facility
Jigawa	Tsangarwa	Budinga MPHCC	Gwaram	Rural	Primary Health Care Facility
Jigawa	Dabi	Dabi Primary Health Care Clinic	Gwiwa	Rural	Primary Health Care Facility
Jigawa	Kore	Fagen Gawo MPHC	Garki	Rural	Primary Health Care Facility
Jigawa	Madobi	Hammayayi BHC	Dutse	Rural	Primary Health Care Facility
Jigawa	Falgeri	Karanjau Health Post	Buji	Rural	Primary Health Care Facility
Jigawa	Kudai	Kudai BHC	Dutse	Urban	Primary Health Care Facility

State	LGA	Facility	Ward	Location	Facility Type
Jigawa	Dabaza	Kurfi HP	Kazaure	Rural	Primary Health Care Facility
Jigawa	Kwalam	KWALAM MPHC	Taura	Urban	Primary Health Care Facility
Jigawa	Mai Aduwa	MAI ADUA BHC	Gagarawa	Rural	Primary Health Care Facility
Jigawa	Muku	Muku BHC	Garki	Rural	Primary Health Care Facility
Jigawa	Kila	Nasarawa Health Post	Gwaram	Rural	Primary Health Care Facility
Jigawa	Idanduna	Rinde BHC	Jahun	Rural	Primary Health Care Facility
Jigawa	Ringim	RURUMA HP	Yankwashi	Rural	Primary Health Care Facility
Jigawa	Bulangu	Sabon SARA BHC	Kafin Hausa	Rural	Primary Health Care Facility
Jigawa	Shabaru	SHABARU BHC	Sule Tankarkar	Rural	Primary Health Care Facility
Jigawa	Ringim	WALAWA MCHC	Ringim	Urban	Primary Health Care Facility
Jigawa	Sakwaya	Warwade HP	Dutse	Rural	Primary Health Care Facility
Jigawa	Karnaya	Wurma BHC	Dutse	Rural	Primary Health Care Facility
Kaduna	LERE	CHC Saminaka	SAMINAKA	Rural	Primary Health Care Facility
Kaduna	KADUNA NORTH	H/C MADARA	SHABA	Urban	Primary Health Care Facility
Kaduna	KAURA	HC ANTURUNG	KAURA	Urban	Primary Health Care Facility
Kaduna	KUDAN	HC Lafiya	GARU	Rural	Primary Health Care Facility
Kaduna	ZARIA	Health Clinic Jagina Tasha	KUFENA	Urban	Primary Health Care Facility
Kaduna	GIWA	HEALTH CLINIC MADARA SARKI	GANGARA	Rural	Primary Health Care Facility
Kaduna	KAURU	Kurmin Ruwa	BADURUM	Rural	Primary Health Care Facility
Kaduna	KAGARKO	PHC Akot	KAGARKO NORTH	Rural	Primary Health Care Facility
Kaduna	KADUNA NORTH	PHC ANG/BARAU	BADARAWA	Urban	Primary Health Care Facility
Kaduna	ZARIA	PHC BABANDODO	LIMANCIN KONA	Urban	Primary Health Care Facility
Kaduna	KAJURU	PHC Buda	BUDA	Rural	Primary Health Care Facility
Kaduna	LERE	PHC Dogondaji	RAMINKURA	Rural	Primary Health Care Facility
Kaduna	JEMA'A	PHC GIDAN WAYA	JAGINDI	Rural	Primary Health Care Facility
Kaduna	IKARA	PHC IKARA	IKARA	Urban	Primary Health Care Facility
Kaduna	JEMA'A	PHC JOS ROAD	GIDAN WAYA	Rural	Primary Health Care Facility
Kaduna	JEMA'A	PHC Kafanchan	KAFANCHAN A	Urban	Primary Health Care Facility
Kaduna	KAGARKO	PHC Kagarko	KAGARKO SOUTH	Rural	Primary Health Care Facility
Kaduna	KAJURU	PHC Kalla	KALLAH	Rural	Primary Health Care Facility
Kaduna	KAJURU	PHC Kasuwa Magani	KASUWAN MAGANI	Rural	Primary Health Care Facility
Kaduna	IKARA	PHC Kaya	JAMPALAN	Rural	Primary Health Care Facility
Kaduna	SOBA	PHC Kinkiba	KINKIBA	Rural	Primary Health Care Facility
Kaduna	SABON GARI	PHC Samaru	SAMARU	Urban	Primary Health Care Facility
Kaduna	ZANGON KATAF	PHC Zankwa	ZONKWA	Urban	Primary Health Care Facility
Kaduna	KAURA	RURAL HOSPITAL KAURA	KAURA	Rural	Primary Health Care Facility
Kano	Ajingi	Ajingi PHC	Ajingi	Rural	Primary Health Care Facility
Kano	Badume	BADUME MODEL	Bichi	Rural	Primary Health Care Facility
Kano	Kwa	Buruntumau Health Post	Dawakin Tofa	Rural	Primary Health Care Facility
Kano	Yallami	Daddo Health Post	Bichi	Rural	Primary Health Care Facility

State	LGA	Facility	Ward	Location	Facility Type
Kano	Unguwar Rmi (KBT)	Dangwaro Health Post	Kumbotso	Urban	Primary Health Care Facility
Kano	Kumurya	Dususu Health Post	Bunkure	Rural	Primary Health Care Facility
Kano	Kurugu	Dutsen Amare Health Post	Karaye	Rural	Primary Health Care Facility
Kano	Danbatta	Fagwalo Health Post	Danbatta	Rural	Primary Health Care Facility
Kano	Gaya North	Gachi Health Post	Gaya	Rural	Primary Health Care Facility
Kano	Yallami	Galaji Health Post	Bichi	Rural	Primary Health Care Facility
Kano	Karmami	Gumawa Health Post	Gabasawa	Rural	Primary Health Care Facility
Kano	Dawaki West	Joben Marke Health Post	Dawakin Tofa	Rural	Primary Health Care Facility
Kano	Bargoni	Kailani Health Post	Kiru	Rural	Primary Health Care Facility
Kano	Kofar Ruwa	Kofar Ruwa Basic Health Clinic	Dala	Urban	Primary Health Care Facility
Kano	Gofon	Makunturi Health Clinic	Bunkure	Rural	Primary Health Care Facility
Kano	Gwarabjawa	Miaganji Health Post	Danbatta	Rural	Primary Health Care Facility
Kano	Chalawa	Rinkusawa Health Post	Kumbotso	Urban	Primary Health Care Facility
Kano	Bunkure	Sabon Ruwa Health Clinic	Bunkure	Rural	Primary Health Care Facility
Kano	Sare Sare	Surfan Health Post	Bagwai	Rural	Primary Health Care Facility
Kano	Yalwa Dala	Yalwa Mailafiya Sanka Health Clinic	Dala	Urban	Primary Health Care Facility
Kano	Yumbu	Yama Kanawa Health Post	Gabasawa	Rural	Primary Health Care Facility
Kano	Yammedi	Yammedi Primary Health Centre	Karaye	Rural	Primary Health Care Facility
Kano	Badume	Yola Health Post (BCH)	Bichi	Rural	Primary Health Care Facility
Kano	Tattarawa	Zaura Health Post	Dawakin Tofa	Rural	Primary Health Care Facility
Katsina	Bindawa	Adarkawa Dispensary	Bindawa	Rural	Primary Health Care Facility
Katsina	Kankara	Badau Health Clinic	Kankara	Rural	Primary Health Care Facility
Katsina	Mani	Baryawa Health Clinic	Mani	Rural	Primary Health Care Facility
Katsina	Malumfashi	Dansarai Health Facility	Malumfashi	Urban	Primary Health Care Facility
Katsina	Mani	Gamda Health Clinic	Mani	Rural	Primary Health Care Facility
Katsina	Malumfashi	Gwanamarde Health Clinic	Malumfashi	Rural	Primary Health Care Facility
Katsina	Batagarawa	Jino MCHC	Batagarawa	Urban	Primary Health Care Facility
Katsina	Bakori	Kabomo Health Clinic	Bakori	Rural	Primary Health Care Facility
Katsina	Kankia	Kafin Dangi MCHC	Kankia	Rural	Primary Health Care Facility
Katsina	Katsina	Kofar Sauri Clinic	Katsina	Urban	Primary Health Care Facility
Katsina	Batagarawa	Kurtufa MCHC	Batagarawa	Urban	Primary Health Care Facility
Katsina	Batagarawa	MAJEN ABDU MCHC	Batagarawa	Urban	Primary Health Care Facility
Katsina	Malumfashi	Mararrabar Kankara Health Clinic	Malumfashi	Rural	Primary Health Care Facility
Katsina	Batagarawa	MCHC Bakiyawa	Batagarawa	Urban	Primary Health Care Facility
Katsina	Kafur	Sabuwa Kasa M.C.H.C	Kafur	Rural	Primary Health Care Facility
Katsina	Katsina	Sabuwar Unguwa HC	Katsina	Urban	Primary Health Care Facility
Katsina	Mani	Samfa HP	Mani	Rural	Primary Health Care Facility

State	LGA	Facility	Ward	Location	Facility Type
Katsina	Kafur	Tabkin Jage Health Facility	Kafur	Rural	Primary Health Care Facility
Katsina	Kankia	Tashar Gamji Health Clinic	Kankia	Rural	Primary Health Care Facility
Katsina	Ingawa	Todai Health Facility	Ingawa	Rural	Primary Health Care Facility
Katsina	Katsina	Wakilin Arewa Health Clinic	Katsina	Urban	Primary Health Care Facility
Katsina	Ingawa	Wanzamai Dispensary	Ingawa	Rural	Primary Health Care Facility
Katsina	Kankia	Yarkutungu Health Clinic	Kankia	Rural	Primary Health Care Facility
Katsina	Kafur	Yartalata Health Facility	Kafur	Rural	Primary Health Care Facility
Kebbi	AREWA DANDI	BACHAKA PHC	BACHAKA	Rural	Primary Health Care Facility
Kebbi	ALEIRO	BAKAMBARA HEALTH CENTRE	ALIERO DANGALADIMA I	Urban	Primary Health Care Facility
Kebbi	AREWA DANDI	BUI COMPREHENSIVE HEALTH CENTER	BUI	Rural	Primary Health Care Facility
Kebbi	BUNZA	DANGALADIMA PHC	BUNZA DANGALADIMA	Rural	Primary Health Care Facility
Kebbi	ARGUNGU	GRA HF	KOKANI SOUTH	Urban	Primary Health Care Facility
Kebbi	YAURI	JIJIMA PHC	JIJIMA	Rural	Primary Health Care Facility
Kebbi	SURU	KWAKARE PRIMARY HEALTH CENTRE	BAKUWAI	Rural	Primary Health Care Facility
Kebbi	DANDI	KWAKWABA PHC	KWAKKWABA	Rural	Primary Health Care Facility
Kebbi	FAKAI	MAHUTA MATERNAL AND CHILD HEALTH CENTER	MAHUTA	Rural	Primary Health Care Facility
Kebbi	AUGIE	PHC BAGURA	YOLA	Rural	Primary Health Care Facility
Kebbi	AUGIE	PHC BAYAWA SHIYAR	BAYAWA NORTH	Rural	Primary Health Care Facility
Kebbi	Wasagu-Danko	PHC DIKENGE	kyabu/Kandu	Rural	Primary Health Care Facility
Kebbi	BAGUDO	PHC GWAMBA	KAOJE GWAMBA	Rural	Primary Health Care Facility
Kebbi	JEGA	PHC JANDUTSI	JANDUTSI BIRNIN MALAM	Rural	Primary Health Care Facility
Kebbi	AUGIE	PHC LUGA MARAFA	TIGGI	Urban	Primary Health Care Facility
Kebbi	KOKO-BESSE	PHC MANYATAFUKKA	LANI SHIBA	Rural	Primary Health Care Facility
Kebbi	AUGIE	PHC MERA	BAGAYE MERA	Rural	Primary Health Care Facility
Kebbi	ZURU	PHC RIKOTO	RIKOTO	Rural	Primary Health Care Facility
Kebbi	ALEIRO	PRIMARY HEALTH CENTRE SABIYAL	SABIYAL	Rural	Primary Health Care Facility
Kebbi	SHANGA	RAHA MATERNAL AND CHILD HEALTH CENTRE	DUGU TSOHO	Rural	Primary Health Care Facility
Kebbi	BIRNIN KEBBI	RANDALI HP	LAGGA	Rural	Primary Health Care Facility
Kebbi	ARGUNGU	SABONGARIN KANTA HF	KOKANI NORTH	Urban	Primary Health Care Facility
Kebbi	WASAGU-DANKO	UNGUWAN KIBIYA DISPENSARY.	AYU	Rural	Primary Health Care Facility
Kebbi	BIRNIN KEBBI	UNGUWAR MIJIN NANA CHC	MAURIDA	Rural	Primary Health Care Facility
Sokoto	YABO	ALKALINE PHC	KILGORI	Rural	Primary Health Care Facility

State	LGA	Facility	Ward	Location	Facility Type
Sokoto	SOKOTO NORTH	ALKAMAWA PHC	MAGAJIN RAFI A	Urban	Primary Health Care Facility
Sokoto	GUDU	BHC YAKA	GWAZANGE	Rural	Primary Health Care Facility
Sokoto	KWARE	COMPREHENSIVE HEALTH CENTER	KWARE	Rural	Primary Health Care Facility
Sokoto	BODINGA	DANCHADI PRIMARY HEALTH CENTRE	DANCHADI	Rural	Primary Health Care Facility
Sokoto	KWARE	DURBAWA PRIMARY HEALTH CARE	DURBAWA	Rural	Primary Health Care Facility
Sokoto	SOKOTO SOUTH	GIDAN DAHALA DISPENSARY	GAGI C	Urban	Primary Health Care Facility
Sokoto	SHAGARI	HORO DAN BARO DISPENSARYE	HORO	Rural	Primary Health Care Facility
Sokoto	KEBBE	JIGIRI DISPENSARY	MARGAI EAST	Rural	Primary Health Care Facility
Sokoto	GADA	KADADIN BUDA PHC	KADADI	Rural	Primary Health Care Facility
Sokoto	GADA	KADADIN MAIDABO PHC	KADADI	Rural	Primary Health Care Facility
Sokoto	GWADABAWA	KATTALA DISPENSARY	ATAKWANYO	Rural	Primary Health Care Facility
Sokoto	WURNO	MODEL PRIMARY HEALTH CARE ACHIDA	ACHIDA	Rural	Primary Health Care Facility
Sokoto	TANGAZA	MOGOHO HEALTH CLINIC	MAGONHO	Rural	Primary Health Care Facility
Sokoto	GWADABAWA	PHC ASARA	ASARA	Rural	Primary Health Care Facility
Sokoto	DANGE-SHUNI	PHC GE ERE	GE'ERE GAJARA	Rural	Primary Health Care Facility
Sokoto	ILLELA	PHC KALMALO	KALMALO	Rural	Primary Health Care Facility
Sokoto	GUDU	PHC KARFEN SARKI	KARFE SARKI	Rural	Primary Health Care Facility
Sokoto	GWADABAWA	PHC MAKINA	MAMMAN SUKA	Rural	Primary Health Care Facility
Sokoto	TANGAZA	RUWA WURI HP	RUWA WURI	Rural	Primary Health Care Facility
Sokoto	SILAME	TANERA PRIMARY HEALTH CARE CENTER	KATAMI SOUTH	Rural	Primary Health Care Facility
Sokoto	BODINGA	TULUWA PHC	TULLUWA	Rural	Primary Health Care Facility
Sokoto	SOKOTO SOUTH	TURAKI PHC	SARKIN ZAMFARA A	Urban	Primary Health Care Facility
Sokoto	KWARE	ZAMAU PHC	TSAKI WALAKHE	Rural	Primary Health Care Facility
Zamfara	BUNGUDU	ASAKO DISP	BELA RWAYYA	Rural	Primary Health Care Facility
Zamfara	MARADUN	DANBAZA HC	DOSARA BIRNIN KAYA	Rural	Primary Health Care Facility
Zamfara	BUNGUDU	DANMARKE PHC	GADA KARAKAI	Rural	Primary Health Care Facility
Zamfara	BUNGUDU	DASHI CLINIC	SAMAWA	Rural	Primary Health Care Facility
Zamfara	MARADUN	DOSARA DISP	DOSARA BIRNIN KAYA	Rural	Primary Health Care Facility
Zamfara	GUSAU	DR KARIMA	TUDUN WADA	Urban	Primary Health Care Facility
Zamfara	GUSAU	GEBA CLINIC	RIJIYA	Rural	Primary Health Care Facility
Zamfara	GUSAU	KARAL CLINIC	WANKE	Rural	Primary Health Care Facility
Zamfara	BUNGUDU	KOTORKOSHI PHC	KOTORKOSH	Rural	Primary Health Care Facility
Zamfara	GUSAU	MADA PHC	MADA	Rural	Primary Health Care Facility
Zamfara	TSAFE	PHC BILBIS	BILBIS	Rural	Primary Health Care Facility
Zamfara	BAKURA	PHC DAMRI	DAMRI	Rural	Primary Health Care Facility
Zamfara	ZURMI	PHC DAURAN	DAURAN BIRNIN TSABA	Rural	Primary Health Care Facility

State	LGA	Facility	Ward	Location	Facility Type
Zamfara	KAURA NAMODA	PHC KASUWAR DAJI	KYAMBARAWA	Rural	Primary Health Care Facility
Zamfara	ANKA	PHC WUYA	WUYA	Rural	Primary Health Care Facility
Zamfara	TSAFE	PHC YANDOTO	YANDOTON DAJI	Rural	Primary Health Care Facility
Zamfara	BUKKUYUM	PHC ZAUMA	ZAUMA	Rural	Primary Health Care Facility
Zamfara	GUSAU	RIJIYA PHC	RIJIYA	Rural	Primary Health Care Facility
Zamfara	GUSAU	SHAGARI PHC	MAYANA	Urban	Primary Health Care Facility
Zamfara	KAURA NAMODA	TUNGAR HARUNA	KURYA	Rural	Primary Health Care Facility
Zamfara	BUNGUDU	TUNGAR MASU	GADA KARAKAI	Rural	Primary Health Care Facility
Zamfara	BUNGUDU	WAZOJI	TOFA	Rural	Primary Health Care Facility
Zamfara	TALATA MAFARA	WCWC T/MAFARA	KAYAYE	Rural	Primary Health Care Facility
Zamfara	BUNGUDU	YARLABE DISP	BELA RWAYYA	Rural	Primary Health Care Facility
**South East**					
Anambra	Awka South	Agulu Health Post	Agulu Awka	Rural	Primary Health Care Facility
Anambra	Idemili North	Abacha PHC	Abacha	Rural	Primary Health Care Facility
Anambra	Nnewi South	Azigbo PHC	Azigbo	Rural	Primary Health Care Facility
Anambra	Dunukofia	Dunukofia Comperehensive HC	Ukpo	Rural	Primary Health Care Facility
Anambra	Ekwusigo	Ibolo HC	Ibolo	Rural	Primary Health Care Facility
Anambra	Ogbaru	Iyiowa PHC	Iyiowa/Od/Ohi	Rural	Primary Health Care Facility
Anambra	Onitsha North	Marine Health Post	Marine & 3-3	Urban	Primary Health Care Facility
Anambra	Nnewi North	Mbanagu PHC	Otolo III	Rural	Primary Health Care Facility
Anambra	Oyi	Oyolu PHC	Nkwelle II	Rural	Primary Health Care Facility
Anambra	Njikoka	PHC Abagana	Abagana IV	Rural	Primary Health Care Facility
Anambra	Idemili South	PHC Akwu Ukwu	Akwu Ukwu	Rural	Primary Health Care Facility
Anambra	Ihiala	PHC Amorka	Amorka	Rural	Primary Health Care Facility
Anambra	Oyi	PHC Awkuzu	Awkuzu	Rural	Primary Health Care Facility
Anambra	Idemili North	PHC Eziowelle	Eziowelle	Rural	Primary Health Care Facility
Anambra	Aguata	PHC Ifite Igboukwu	Igboukwu II	Rural	Primary Health Care Facility
Anambra	Ihiala	PHC Mbosi	Mbosi	Rural	Primary Health Care Facility
Anambra	Awka South	PHC Nibo	Nibo II	Rural	Primary Health Care Facility
Anambra	Anaocha	PHC Nri I	Nri I	Rural	Primary Health Care Facility
Anambra	Aguata	PHC Ozara Akukwa	Umuchu I	Rural	Primary Health Care Facility
Anambra	Onitsha South	Redemption HP	Fegge III	Urban	Primary Health Care Facility
Anambra	Nnewi North	Uru PHC	Umudim	Rural	Primary Health Care Facility
Anambra	Onitsha North	Basic HC	Ogbemuonicha	Urban	Primary Health Care Facility
Anambra	Idemili South	Ebenesi	Nibo I	Rural	Primary Health Care Facility
Anambra	Onitsha South	ST. John De Baptist	Fegge III	Urban	Primary Health Care Facility
Imo	ORU-WEST	AJI PHC	AJI	Rural	Primary Health Care Facility
Imo	ORLU	AMAIFEKE EZIOHA	AMAIFEKE EZIOHA	Rural	Primary Health Care Facility
Imo	NKWERRE	AMAOKPARA HC	AMAOKPARA	Rural	Primary Health Care Facility
Imo	ABOH MBAISE	AMUZU HEALTH CENTRE	AMUZU	Rural	Primary Health Care Facility
Imo	OW. MUNICIPAL	AREA M CHC	MUNICIPAL	Urban	Primary Health Care Facility
Imo	ISU	EBENATOR PHC	EBENATO	Rural	Primary Health Care Facility

State	LGA	Facility	Ward	Location	Facility Type
Imo	ABOH MBAISE	IBEKU PHC	IBEKU	Rural	Primary Health Care Facility
Imo	OKIGWE	IHUBE Health Centre	IHUBE 2	Rural	Primary Health Care Facility
Imo	OW. MUNICIPAL	IMMUNIZATION UNIT STAFF CLINIC	MUNICIPAL	Urban	Primary Health Care Facility
Imo	ABOH MBAISE	LORJI HEALTH CENTRE	LORJI	Rural	Primary Health Care Facility
Imo	ORLU	MCH ORLU	AMAIFEKE OFEAHIA	Rural	Primary Health Care Facility
Imo	ORLU	MPHCC OFEHIA AMAIFEKE	AMAIFEKE OFEAHIA	Urban	Primary Health Care Facility
Imo	IKEDURU	OGADA Health Centre	ATTA 11	Rural	Primary Health Care Facility
Imo	ORLU	OHAEKE HEALTH CLINIC	OKPORO OHAEKE	Rural	Primary Health Care Facility
Imo	EHIME MBANO	OKUNRAYE PHC	UMUEZE 11	Rural	Primary Health Care Facility
Imo	ONUIMO	OKWE PHC	EZIAMA	Rural	Primary Health Care Facility
Imo	ORLU	OWERRE-EBEIRI PHC	OWERE EBEIRI	Rural	Primary Health Care Facility
Imo	ORU-WEST	OZARA HEALTH CENTRE	OZARA	Rural	Primary Health Care Facility
Imo	NKWERRE	UMUDI HEALTH CENTRE	UMUDI	Rural	Primary Health Care Facility
Imo	EHIME MBANO	UMUDURU EGWELLE	UMUEZE 1	Rural	Primary Health Care Facility
Imo	ONUIMO	UMUDURU PHC	OFEAHIA	Rural	Primary Health Care Facility
Imo	EHIME MBANO	UMUNAKANU HEALTH CENTRE	UMUNAKANU	Rural	Primary Health Care Facility
Imo	OW. MUNICIPAL	UMUODU Health Centre	MUNICIPAL	Urban	Primary Health Care Facility
Imo	ISU	UMUOKWARA HEALTH POST	UMUNDUGBA	Rural	Primary Health Care Facility
**South South**					
Bayelsa	Yenegoa	Aguduma Epie	Epie 1	Urban	Primary Health Care Facility
Bayelsa	Yenegoa	Amarata PHC	Epie 3	Urban	Primary Health Care Facility
Bayelsa	Yenegoa	Basic Health Center Akaba	Atisa 3	Urban	Primary Health Care Facility
Bayelsa	Yenegoa	Basic Health Center Famgbe	Atisa 2	Urban	Primary Health Care Facility
Bayelsa	Southern Ijaw	CHC Amassoma	Amassoma 1	Rural	Primary Health Care Facility
Bayelsa	Nembe	CHC Bassambiri	Bassambiri	Rural	Primary Health Care Facility
Bayelsa	Nembe	CHC Nembe	Ogbolomabiri	Rural	Primary Health Care Facility
Bayelsa	Brass	CHC Twon Brass	Brass 1	Rural	Primary Health Care Facility
Bayelsa	Yenegoa	Comprehensive Health Center Azikoro	Epie 3	Urban	Primary Health Care Facility
Bayelsa	Brass	Diema PHC	Odioma	Rural	Primary Health Care Facility
Bayelsa	Yenegoa	FSP Clinic	Atisa 1	Urban	Primary Health Care Facility
Bayelsa	Kolokuma/Opokuma	Health Centre Kalama	Sampou/Kalama	Rural	Primary Health Care Facility
Bayelsa	Brass	Imbikiri PHC	Brass 2	Rural	Primary Health Care Facility
Bayelsa	Yenegoa	Nedugo PHC	Gbarain 1	Urban	Primary Health Care Facility
Bayelsa	Yenegoa	Opolo HC	Epie 2	Urban	Primary Health Care Facility
Bayelsa	Ogbia	Oruma PHC	Otuasega	Rural	Primary Health Care Facility
Bayelsa	Ogbia	Otaeme PHC	Otaeme	Rural	Primary Health Care Facility

State	LGA	Facility	Ward	Location	Facility Type
Bayelsa	Ogbia	Otuasega PHC	Otuasega	Rural	Primary Health Care Facility
Bayelsa	Southern Ijaw	PHC Anyama	Oporoma 2	Rural	Primary Health Care Facility
Bayelsa	Kolokuma/Opokuma	Phc Kaiama	Kaiama	Rural	Primary Health Care Facility
Bayelsa	Kolokuma/Opokuma	Phc Odi	Odi Central	Rural	Primary Health Care Facility
Bayelsa	Ogbia	PHC OGBIA TOWN	Ogbia-Ward 1	Rural	Primary Health Care Facility
Bayelsa	Ogbia	Phc Otuoke	Otueke-Ward 13	Rural	Primary Health Care Facility
Bayelsa	Kolokuma/Opokuma	PHC staff clinic	Kaiama/Olobiri	Rural	Primary Health Care Facility
Rivers	AKUKUTORU	ABONNEMA GENERAL HOSPITAL	AKUKUTORU13 WARD	Rural	Seconday Health Care Facility
Rivers	TAI	CHC OYIGBO	TAI5 WARD	Rural	Primary Health Care Facility
Rivers	OBIO/AKPOR	College of Health Science HC	OBIOAKPOR10 WARD	Urban	Primary Health Care Facility
Rivers	OKRIKA	George Ama MPHC	OKRIKA8 WARD	Rural	Primary Health Care Facility
Rivers	AKUKUTORU	MPHC ABONNEMA	AKUKUTORU1 WARD	Rural	Primary Health Care Facility
Rivers	EMUOHA	MPHC AKPABU	EMUOHA10 WARD	Rural	Primary Health Care Facility
Rivers	IKWERRE	MPHC APANI	IKWERRE6 WARD	Rural	Primary Health Care Facility
Rivers	KHANA	MPHC BOTEM	KHANA17 WARD	Rural	Primary Health Care Facility
Rivers	PORT HARCOURT	MPHC BUNDU	PORTHARTCOURT5 WARD	Urban	Primary Health Care Facility
Rivers	ELEME	MPHC ETEO	ELEME6 WARD	Rural	Primary Health Care Facility
Rivers	EMUOHA	MPHC IBAA	EMUOHA6 WARD	Rural	Primary Health Care Facility
Rivers	IKWERRE	MPHC IGWURUTA	IKWERRE8 WARD	Rural	Primary Health Care Facility
Rivers	OKRIKA	MPHC OKOCHIRI	OKRIKA5 WARD	Rural	Primary Health Care Facility
Rivers	PORT HARCOURT	MPHC POTTS JOHNSON	PORTHARTCOURT5 WARD	Urban	Primary Health Care Facility
Rivers	OBIO/AKPOR	MPHC RUMUIGBO	OBIOAKPOR12 WARD	Urban	Primary Health Care Facility
Rivers	EMUOHA	MPHC RUMUJI	EMUOHA13 WARD	Rural	Primary Health Care Facility
Rivers	OBIO/AKPOR	MPHC RUMUOLUMENI	OBIOAKPOR17 WARD	Urban	Primary Health Care Facility
Rivers	EMUOHA	NDELE MODEL PRIMARY HC	EMUOHA7 WARD	Rural	Primary Health Care Facility
Rivers	AKUKUTORU	OBANOMA MPHC	AKUKUTORU14 WARD	Rural	Primary Health Care Facility
Rivers	EMUOHA	ODUOHA MPHC	EMUOHA3 WARD	Rural	Primary Health Care Facility
Rivers	ELEME	OGALE MPHC	ELEME3 WARD	Urban	Primary Health Care Facility
Rivers	IKWERRE	OMAGWA MPHC	IKWERRE9 WARD	Rural	Primary Health Care Facility
Rivers	OBIO/AKPOR	OZUOBA MPHC	OBIOAKPOR16 WARD	Urban	Primary Health Care Facility
Rivers	OYIGBO	PHC BOROBARA	OYIGBO8 WARD	Urban	Primary Health Care Facility
**South West**					
Ekiti	OYE	BASIC H C AYEGBAJU	AYEGBAJU	Rural	Primary Health Care Facility
Ekiti	OYE	BHC ARE	ILUPEJU II	Rural	Primary Health Care Facility
Ekiti	IREPODUN/IFELODUN	BHC AYEGBAJU	IGEDE B	Rural	Primary Health Care Facility
Ekiti	IREPODUN/IFELODUN	BHC IFOFIN	IWOROKO	Rural	Primary Health Care Facility
Ekiti	GBONYIN	BHC IGBARA ODO	IJAN	Rural	Primary Health Care Facility

State	LGA	Facility	Ward	Location	Facility Type
Ekiti	EFON	BHC IJAN	ILORO	Rural	Primary Health Care Facility
Ekiti	ISE/ORUN	BHC IJESA ISU	ERINWA I	Rural	Primary Health Care Facility
Ekiti	IJERO	BHC IKOGOSI	EKAMETA	Rural	Primary Health Care Facility
Ekiti	EKITI WEST	BHC ILORO	IKOGOSI	Rural	Primary Health Care Facility
Ekiti	ADO-EKITI	BHC ILUPEJU	ODO ADO	Urban	Primary Health Care Facility
Ekiti	EKITI SOUTH WEST	BHC IWOROKO	ODO IGBARA	Rural	Primary Health Care Facility
Ekiti	EKITI WEST	BHC JERO OMUO	ARAMOKO II	Rural	Primary Health Care Facility
Ekiti	ADO-EKITI	BHC ODO ADO	OPOPOGBORO	Urban	Primary Health Care Facility
Ekiti	EKITI EAST	BHC OKE OJA	IJERO OMUO	Rural	Primary Health Care Facility
Ekiti	EMURE	BHC OPOPO	OWODE OSE	Rural	Primary Health Care Facility
Ekiti	ADO-EKITI	BHC OWODE	OKEYIMI	Urban	Primary Health Care Facility
Ekiti	EKITI SOUTH WEST	CHC ADIN	ADIN	Rural	Primary Health Care Facility
Ekiti	IKERE	CHC ARA	ATIBA/AFIN	Rural	Primary Health Care Facility
Ekiti	ILEJEMEJE	CHC ATIBA	IYE	Rural	Primary Health Care Facility
Ekiti	MOBA	CHC IYE	OTUN III	Rural	Primary Health Care Facility
Ekiti	IKERE	CHC OTUN	ARE ARAROMI	Rural	Primary Health Care Facility
Ekiti	IJERO	CHC USI	ODO-OWA	Rural	Primary Health Care Facility
Ekiti	IKOLE	FSP CLINIC	IJESA ISU	Rural	Primary Health Care Facility
Ekiti	IJERO	ODO OWA BHC	ODO-OWA	Rural	Primary Health Care Facility
Lagos	IKEJA	Aguda PHC	AGUDA	Urban	Primary Health Care Facility
Lagos	MUSHIN	Ajeabo PHC	IGBEHINADUN	Urban	Primary Health Care Facility
Lagos	ALIMOSHO	Akinogun PHC	MOSAN AKINOGUN	Urban	Primary Health Care Facility
Lagos	ALIMOSHO	Akinyele PHC	ABESAN 2	Urban	Primary Health Care Facility
Lagos	KOSOFE	Alapere PHC	AGIDI OSHOGUN	Urban	Primary Health Care Facility
Lagos	SURULERE	Anjorin PHC	MUNIRU BARUWA	Urban	Primary Health Care Facility
Lagos	L/ISLAND	Araromi PHC	ARAROMI ODO	Urban	Primary Health Care Facility
Lagos	IKEJA	Asiwaju Bola Ahmed Tinubu PHC	ALADE	Urban	Primary Health Care Facility
Lagos	SURULERE	Baruwa PHC	SANUSI	Urban	Primary Health Care Facility
Lagos	AGEGE	Dopemu PHC	ISALEOJA	Urban	Primary Health Care Facility
Lagos	EPE	Epe PHC	BADO	Rural	Primary Health Care Facility
Lagos	IKEJA	Ikeja PHC	ANIFOWOSHE	Urban	Primary Health Care Facility
Lagos	KOSOFE	Ikosi PHC	IKOSI OKE	Urban	Primary Health Care Facility
Lagos	L/MAINLAND	Iwaya PHC	SALAMI BAYEWUNMI	Urban	Primary Health Care Facility
Lagos	OSHODI	Jakande PHC	OKE AFA	Urban	Primary Health Care Facility
Lagos	L/MAINLAND	Koola Osho PHC	ABULE-OJA	Urban	Primary Health Care Facility
Lagos	IBEJU	Leki PHC	LEKKI 1	Rural	Primary Health Care Facility
Lagos	KOSOFE	Mende PHC	MENDE	Urban	Primary Health Care Facility
Lagos	IFAKO	Ojokoro PHC	IJAIYE OJOKORO	Urban	Primary Health Care Facility
Lagos	IBEJU	OKUNRAYE PHC	LEKKI 2	Rural	Primary Health Care Facility
Lagos	L/ISLAND	Onikan PHC	ARAROMI ODO	Urban	Primary Health Care Facility
Lagos	OSHODI	Oshodi PHC	IGBEYINADUN	Urban	Primary Health Care Facility
Lagos	IBEJU	Palm Avenue PHC	LEKKI 2	Rural	Primary Health Care Facility

State	LGA	Facility	Ward	Location	Facility Type
Lagos	ALIMOSHO	Rauf Aregbesola PHC	OKUNOLA	Urban	Primary Health Care Facility

Annex S3. Module B: Health Care Worker Survey

Instructions:
This section will gather basic information about the facility and service delivery practices and should be directed to ***an immunization health care worker****.*

Read aloud to the respondent:
I WOULD LIKE TO ASK YOU SOME BASIC INFORMATION ABOUT THIS FACILITY AND YOUR IMMUNIZATION SESSIONS. IF AT ANY POINT YOU ARE UNSURE OF THE CORRECT RESPONSE YOU MAY ASK ONE OF YOUR COLLEAGUES OR SKIP THE QUESTION.

SECTION I: FACILITY SERVICES AND OPERATIONS			
**NO.**	**QUESTION**	**RESPONSE CODE**	**SKIP**
FS1	WHAT IS YOUR POSITION AT THIS FACILITY?	---	
FS2	HOW MANY DAYS PER WEEK IS THIS FACILITY OPEN?	# of open days per week........................ ___	
FS3	ON AVERAGE, HOW MANY HOURS PER DAY IS THIS FACILITY OPEN?<br><br>*Special codes:*<br>*24 = 24 hours per day*<br>*97 = Refused*<br>*98 = Don't know*	# of open hours per day................. ___ ___	
FS4	IN YOUR CATCHMENT AREA, ARE THERE ANY HARD-TO-REACH OR SPECIAL POPULATIONS?	A. Yes<br>B. No<br>C. Don't know	A → FS5<br>B → FS6<br>C → FS6
FS5	WHAT KIND OF HARD-TO-REACH OR SPECIAL POPULATIONS ARE IN THE AREA?	A. nomadic<br>B. semi-nomadic<br>C. migrants<br>D. minority groups<br>E. non-compliance / vaccine refusal groups<br>F. geographically hard-to-reach<br>G. seasonally hard-to-reach<br>H. Other (specify):_________________________	

FS6	HOW DO YOU DETERMINE THE NUMBER OF IMMUNIZATION SESSIONS YOU HOLD PER MONTH?	A. Vaccine availability<br>B. Number of beneficiaries<br>C. Number of nurses<br>D. Don't know<br>E. Other (specify):________________________	
FS7	IN THE PAST 1 MONTH, HAVE YOU EXPERIENCED STOCK-OUT FOR ANY OF THE FOLLOWING VACCINES?<br>[A] BCG?<br>[B] OPV?<br>[C] HEPB?<br>[D] PENTA?<br>[E] PCV?<br>[F] IPV<br>[G] MEASLES?<br>[H] YELLOW FEVER?<br>[I] TD	Yes No Ref DK<br>A. BCG 1 2 7 8<br>B. OPV 1 2 7 8<br>C. HEPB 1 2 7 8<br>D. PENTA 1 2 7 8<br>E. PCV 1 2 7 8<br>F. IPV 1 2 7 8<br>G. Measles 1 2 7 8<br>H. Yellow Fever 1 2 7 8<br>I. TD 1 2 7 8	

FS8	HOW MANY STAFF PARTICIPATED IN TODAY'S IMMUNIZATION SESSION IN THIS FACILITY?<br><br>*Special codes:*<br>*97 = Refused*<br>*98 = Don't know*	# of staff assigned to immunization work<br>...___ ___	
FS9	DID ANY HEALTH WORKER AT THE FACILITY RECEIVE TRAINING ON IMMUNIZATION SERVICES IN THE LAST ONE YEAR? VACCINE MANAGEMENT TRAINING, ROUTINE IMMUNIZATION, VACCINE WASTE MANAGEMENT, HMIS?<br><br>[A] VACCINES MANAGEMENT TRAINING?<br><br>[B] ROUTINE IMMUNIZATION?<br><br>[C] VACCINE WASTE MANAGEMENT?<br><br>[D] HMIS?<br><br>[X] OTHER TRAINING SPECIFIC TO IMMUNIZATION<br><br>[________________________________]<br>*(specify)*	Yes No Ref DK<br><br>A. VMI 1 2 7 8<br><br>B. R.I. 1 2 7 8<br><br>C. VWM 1 2 7 8<br><br>D. HMIS 1 2 7 8<br><br>X. Other 1 2 7 8	

SECTION 2: Service Delivery Practices			
**NO.**	**QUESTION**	**RESPONSE CODE**	**SKIP**
SD1	IN THE PAST THREE MONTHS HAVE YOU HAD TO THROW AWAY ANY UNOPENED VACCINES?	Yes.. 1<br>No.. 2<br>Refused.. 7<br>Don't know... 8	1 → SD2<br>2 → SD3<br>7 → SD3<br>8 → SD3

SD2	IN TOTAL, HOW MANY VIALS OF EACH VACCINE WERE THROWN OUT IN THE PAST 3 MONTHS?<br><br>[A] BCG?<br><br>[B] OPV?<br><br>[C] HEPB?<br><br>[D] PENTA?<br><br>[E] PCV?<br><br>[F] IPV<br><br>[G] MEASLES?<br><br>[H] YELLOW FEVER?<br><br>[I] TD<br><br>*Special codes:*<br>*97 = Refused*<br>*98 = Don't know*	A. BCG.. __ __ __<br><br>B. OPV .... __ __ __<br><br>C. HepB.. __ __ __<br><br>D. PENTA .... __ __ __<br><br>E. PCV... __ __ __<br><br>F. IPV .... __ __ __<br><br>G. MEASLES................................ __ __ __<br><br>H. Yellow Fever .... __ __ __<br><br>I. TD .... __ __ __	
SD3	HOW MANY DOSES PER VIAL ARE THERE IN THIS FACILITY FOR THE FOLLOWING VACCINES?<br><br>[A] BCG?<br><br>[B] OPV?<br><br>[C] HEPB?<br><br>[D] PENTA?<br><br>[E] PCV?<br><br>[F] IPV<br><br>[G] MEASLES?<br><br>[H] YELLOW FEVER?<br><br>[I] TD<br><br>*Special codes:*<br>*97 = Refused*<br>98 = *Don't know*	A. BCG.. __ __ __<br><br>B. OPV .... __ __ __<br><br>C. HepB.. __ __ __<br><br>D. PENTA .... __ __ __<br><br>E. PCV... __ __ __<br><br>F. IPV .... __ __ __<br><br>G. MEASLES................................ __ __ __<br><br>H. Yellow Fever .... __ __ __<br><br>I. TD .... __ __ __	
SD4	AT THIS FACILITY, IF A VIAL OF OPV IS NOT FINISHED DURING AN IMMUNIZATION SESSION, DO YOU USE IT AT ANOTHER SESSION?	A. Yes<br>B. No<br>C. Don't know	
SD5	ARE YOU FAMILIAR WITH THE "OPEN VIAL POLICY"?	A. Yes<br>B. No<br>C. Don't know	A → SD6<br>B → SD7<br>C → SD7

SD6	For which vaccines does the "open vial policy" apply?	A. BCG<br>B. OPV<br>C. PENTA<br>D. HepB<br>E. Measles<br>F. Yellow Fever<br>G. TD<br>H. IPV<br>I. Don't know	
SD7	If previously discussed conditions are met, for how long can an open vaccine vial (e.g., DPT) be used for future sessions?	A ________________ number of week(s)<br>B. Don't know	
SD8	What conditions must be met before you reuse a vaccine per the open multi-dose vial policy?	A. expiry date has not passed<br>B. vaccines are stored under appropriate cold chain conditions<br>C. vaccine vial septum has not been submerged in water<br>D. Aseptic technique has been used to withdraw all doses<br>E. Vaccine vial monitor (VVM) in stage 1 or 2<br>F. Don't know	
SD9	Have you ever received special training on OPV administrations	Yes.......... 1<br>No.......... 2<br>Refused.......... 7<br>Don't know.......... 8	
SD10	Are there any challenges with administering OPV?	Yes.......... 1<br>No.......... 2<br>Refused.......... 7<br>Don't know.......... 8	1 → SD7<br>2 → SD8<br>7 → SD8<br>8 → SD8
SD11	What are the challenges with OPV?	Administration..........A<br>Reconstitution..........B<br>Refused..........X<br>Don't know..........Y<br>Other (specify)	
SD12	Select the statement you agree with most…	OPV is harder to give than PCV.......... 1<br>OPV is easier to give than PCV.......... 2<br>OPV and PCV are the same to administer. 3	

SD13	WHAT DO YOU THINK IS THE BEST NUMBER OF DOSES TO HAVE FOR OPV /ORAL VACCINES?<br><br>*Special codes:*<br>*96 = No preference*<br>*97 = Refused*<br>*98 = Don't know*	# of doses per vial.............................. __ __	1-95 → SD9<br>2 → SD10<br>7 → SD10<br>8 → SD10
SD14	WHY DO YOU THINK THIS IS THE BEST NUMBER?	_	
SD15	WHAT DO YOU THINK IS THE BEST NUMBER OF DOSES TO HAVE FOR INJECTIBLE VACCINES (E.G. PENTA, PCV)?<br><br>*Special codes:*<br>*96 = No preference*<br>*97 = Refused*<br>*98 = Don't know*	# of doses per vial.............................. __ __	1 - 95 → SD11<br>96 → SD12<br>97 → SD12<br>98 → SD12
SD16	WHY DO YOU THINK THIS IS THE BEST NUMBER?	_	
SD17	AT THE END OF A FIXED SESSION, DO YOU PUT ANY VACCINES BACK IN THE FRIDGE TO USE DURING THE NEXT SESSION?	Yes.. 1<br>No... 2<br>Refused.. 7<br>Don't know.. 8	1-> SD13<br>2 -> SD14<br>3 -> SD14<br>4 -> SD14

SD18	DO YOU PUT ANY OF THE FOLLOWING VACCINES BACK IN THE FRIDGE? (CIRCLE ALL THAT APPLY)<br><br>[A] BCG?<br><br>[B] OPV?<br><br>[C] HEPB?<br><br>[D] PENTA?<br><br>[E] PCV?<br><br>[F] IPV<br><br>[G] MEASLES?<br><br>[H] YELLOW FEVER?<br><br>[I] TD		Yes	No	Ref	DK	
		A. BCG	1	2	7	8	
		B. OPV	1	2	7	8	
		C. HEPB	1	2	7	8	
		D. PENTA	1	2	7	8	
		E. PCV	1	2	7	8	
		F. IPV	1	2	7	8	
		G. Measles	1	2	7	8	
		H. Yellow Fever	1	2	7	8	
		I. TD	1	2	7	8	

SD19	HOW MANY CHILDREN NEED TO BE PRESENT IN ORDER TO OPEN A NEW VIAL OF EACH VACCINE?<br><br>[A] BCG?<br>[B] OPV?<br>[C] HEPB?<br>[D] PENTA?<br>[E] PCV?<br>[F] IPV<br>[G] MEASLES?<br>[H] YELLOW FEVER?<br>[I] TD<br><br>*Special codes:*<br>*97 = Refused*<br>*98 = Don't know*	A. BCG.. __ __ __<br>B. OPV .... __ __ __<br>C. HepB.. __ __ __<br>D. PENTA .... __ __ __<br>E. PCV.. __ __ __<br>F. IPV .... __ __ __<br>G. MEASLES................................ __ __ __<br>H. Yellow Fever .... __ __ __<br>I. TD .... __ __ __	
SD20	AT THE END OF AN OUTREACH SESSION, WHAT DO YOU DO WITH THE VACCINE?	Return to health facility for storage.......... 1<br>Discard vaccine .... 2<br>Refused.. 7<br>Don't know... 8<br>Other (specify)	
SD21	IF OPENED VACCINES ARE DISCARD AT THE END OF AN OUTREACH SESSION, CHOOSE THE VACCINES THAT APPLY?<br><br>[A] BCG?<br>[B] OPV?<br>[C] HEPB?<br>[D] PENTA?<br>[E] PCV?<br>[F] IPV<br>[G] MEASLES?<br>[H] YELLOW FEVER?<br>[I] TD	Yes No Ref DK<br><br>A. BCG.. __ __ __<br>B. OPV .... __ __ __<br>C. HepB.. __ __ __<br>D. PENTA .... __ __ __<br>E. PCV.. __ __ __<br>F. IPV .... __ __ __<br>G. MEASLES................................ __ __ __<br>H. Yellow Fever .... __ __ __<br>I. TD .... __ __ __	

MODULE c: ENDING THE SURVEY

NO.	QUESTION	RESPONSE CODE	SKIP
RESULT	*Record the result of the observation.*	Session completed .... 1<br>Session completed partially complete ...... 2<br>Refused/Did not consent........................... 3<br>Other (*specify*) 4	
